# PRMT5 Interacting Partners and Substrates in Oligodendrocyte Lineage Cells

**DOI:** 10.3389/fncel.2022.820226

**Published:** 2022-03-17

**Authors:** David K. Dansu, Jialiang Liang, Ipek Selcen, Haiyan Zheng, Dirk F. Moore, Patrizia Casaccia

**Affiliations:** ^1^Neuroscience Initiative, Advanced Science Research Center, CUNY, New York, NY, United States; ^2^Graduate Program in Biochemistry, The Graduate Center of the City University of New York, New York, NY, United States; ^3^Department of Neuroscience, Icahn School of Medicine at Mount Sinai, New York, NY, United States; ^4^Graduate School of Biomedical Sciences, Icahn School of Medicine at Mount Sinai, New York, NY, United States; ^5^Center for Advanced Biotechnology and Medicine, Piscataway, NJ, United States; ^6^Department of Biochemistry and Molecular Biology, Robert-Wood Johnson Medical School, Rutgers Biomedical and Health Sciences, Piscataway, NJ, United States; ^7^Department of Biostatistics, School of Public Health, Rutgers, The State University of New Jersey, Piscataway, NJ, United States

**Keywords:** brain, arginine methylation, RNA processing, epigenetics, iTRAQ

## Abstract

The protein arginine methyl transferase PRMT5 is an enzyme expressed in oligodendrocyte lineage cells and responsible for the symmetric methylation of arginine residues on histone tails. Previous work from our laboratory identified PRMT5 as critical for myelination, due to its transcriptional regulation of genes involved in survival and early stages of differentiation. However, besides its nuclear localization, PRMT5 is found at high levels in the cytoplasm of several cell types, including oligodendrocyte progenitor cells (OPCs) and yet, its interacting partners in this lineage, remain elusive. By using mass spectrometry on protein eluates from extracts generated from primary oligodendrocyte lineage cells and immunoprecipitated with PRMT5 antibodies, we identified 1196 proteins as PRMT5 interacting partners. These proteins were related to molecular functions such as RNA binding, ribosomal structure, cadherin and actin binding, nucleotide and protein binding, and GTP and GTPase activity. We then investigated PRMT5 substrates using iTRAQ-based proteomics on cytosolic and nuclear protein extracts from CRISPR-PRMT5 knockdown immortalized oligodendrocyte progenitors compared to CRISPR-EGFP controls. This analysis identified a similar number of peptides in the two subcellular fractions and a total number of 57 proteins with statistically decreased symmetric methylation of arginine residues in the CRISPR-PRMT5 knockdown compared to control. Several PRMT5 substrates were in common with cancer cell lines and related to RNA processing, splicing and transcription. In addition, we detected ten oligodendrocyte lineage specific substrates, corresponding to proteins with high expression levels in neural tissue. They included: PRC2C, a proline-rich protein involved in methyl-RNA binding, HNRPD an RNA binding protein involved in regulation of RNA stability, nuclear proteins involved in transcription and other proteins related to migration and actin cytoskeleton. Together, these results highlight a cell-specific role of PRMT5 in OPC in regulating several other cellular processes, besides RNA splicing and metabolism.

## Introduction

The protein arginine methyltransferases (PRMTs) family of enzymes catalyzes the transfer of methyl groups from S-adenosyl methionine (SAM) to guanidine nitrogen of peptidyl arginine residues in mammals (Bedford and Clarke, 2009) and their role in oligodendrocyte lineage cells has been recently highlighted (Blanc and Richard, 2017). PRMTs can be grouped into three categories, based on their final methylarginine products. While type III PRMTs (including PRMT-7 and 9) (Feng et al., 2013; Yang Y. et al., 2015) only generate monomethylarginines (MMA) (Zurita-Lopez et al., 2012), type I PRMTs (including PRMT-1, -2, -3, -4/CARM1, -6, and 8) catalyze the conversion of MMA to asymmetric dimethylarginines (ADMA) while type II PRMTs (including PRMT-5 and -9), generate symmetric dimethylarginines (SDMA) (Tewary et al., 2019). The functional importance of the steric localization of methyl groups on specific arginine residues on the tails of histone H3 (H3R2 and H3R8) and that of histone H4 (H4R3), has been highlighted (Wang et al., 2001; Pal et al., 2004). The symmetric or asymmetric deposition on the same histone R residue results in opposing functions, either promoting or repressing transcription, depending on the position of the arginines and of the adjacent amino acids (Bedford and Richard, 2005; Litt et al., 2009). Besides transcriptional regulation (Kleinschmidt et al., 2008), arginine methylated residues have been shown to play important roles in RNA metabolism and DNA repair (Bedford and Clarke, 2009), thereby affecting diverse cellular functions, including survival and proliferation (Hua et al., 2020; Tanaka et al., 2020).

Among the PRMTs, the type II PRMT5 is highly expressed in the brain and enriched in oligodendrocyte lineage cells (Huang et al., 2011; Zhang et al., 2014; Tasic et al., 2016). Previous work from our lab and others has highlighted the importance of post-translational modification of amino acids on histone tails in regulating the behavior of oligodendrocyte progenitors Liu et al., 2015, 2016; Moyon et al., 2016; Pruvost and Moyon, 2021). For instance, we previously reported the existence of an interesting cross-talk between PRMT5-mediated symmetric dimethylation of arginine residue R3 and acetylation of lysine residue K5 on histone H4, as critical for oligodendrocyte progenitor survival and differentiation (Scaglione et al., 2018). OPCs lacking *Prmt5* failed to differentiate *in vitro* (Huang et al., 2011), an event attributed to the persistent expression of differentiation inhibitors (*Id2* and *Id4*) (Huang et al., 2011) and more recently attributed to broader effects of PRMT5 loss of function on increased histone acetylation and P53-mediated apoptosis leading to defective myelination (Scaglione et al., 2018), a finding independently validated by a different group (Calabretta et al., 2018). However, PRMT5 is predominantly found in the cytoplasm of OPC and of several other cell types, such as cancer cells (Gu et al., 2012) and stem cells (Koh et al., 2015). In these other cell types, cytosolic PRMT5 has been shown to regulate several aspects of RNA metabolism, including spliceosome assembly, through methylation of Sm proteins (Meister and Fischer, 2002; Gonsalvez et al., 2007), RNA splicing, through methylation of the splicing regulator SRSF1 (Radzisheuskaya et al., 2019), ribosome biogenesis, through methylation of RPS10 (Gao et al., 2017) and RNA transport, *via* arginine methylation of hnRNPA1 (Ren et al., 2010). Cytoplasmic PRMT5 has also been shown to methylate several transcription factors including sterol regulatory element-binding transcription factor 1 (SREBP1) and programmed cell death protein 4 (PDCD4), leading to their nuclear translocation and regulation of their targets (Liang et al., 2021).

Therefore, many of the previous studies focused on PRMT5 as critical regulator of RNA biology, while our previous study addressed the nuclear function of PRMT5, despite its predominant cytosolic localization in OPCs. In this study we use mass spectrometry-based proteomic approaches and iTRAQ labeling of nuclear and cytosolic protein extracts, to broaden our understanding of the role of PRMT5 in OPC by identifying its protein interactors and substrates in oligodendrocyte lineage cells.

## Materials and Methods

### Primary Oligodendrocyte Progenitor Cell Isolation

Primary mouse OPCs were generated from the cortices of C57BL/6 mice, purchased from Jackson Laboratories and euthanized at postnatal day 7 according to IACUC approved protocols at Icahn School of Medicine. Progenitors were immunopanned with a rat anti-mouse CD140a antibody (BD Bioscience, 558774), as previously described (J. Liu et al., 2015). The isolated cells were then cultured in SATO medium (Dulbecco’s modified Eagle’s medium (DMEM), 100 μg/mL BSA, 100 μg/mL apo-transferrin, 16 μg/mL putrescine, 62.5 ng/mL progesterone, 40 ng/mL selenium, 5 μg/mL insulin, 1 mM sodium pyruvate, 5 μg/ml N-acetyl-cysteine, 10 ng/ml biotin, 5 μM forskolin, B27 Supplement and Trace Element B). Progenitors were plated on PDL coated dishes and cultured in SATO medium supplemented either with Platelet-Derived Growth Factor-AA (PDGFA) (10 ng/ml) and basic Fibroblast Growth Factor (bFGF) (20 ng/ml) or supplemented with 60 nM of Triiodothyronine (T3) in the absence of growth factors.

### Immortalized Oligodendrocyte Progenitors (Oli-neu)

Immortalized oligodendrocyte progenitors were received as a gift from Jackie Trotter (Jung et al., 1995) and were cultured on poly-D-lysine coated plates in media (Dulbecco’s modified Eagle’s medium (DMEM), 100 μg/mL BSA, 100 μg/mL apo-transferrin, 16 μg/mL putrescine, 62.5 ng/mL progesterone, 40 ng/mL selenium, 5 μg/mL insulin, 1 mM sodium pyruvate, 5 μg/ml N-acetyl-cysteine, 10 ng/ml biotin, 5 μM forskolin, B27 Supplement, Trace Element B and 1% horse serum).

### Lenti-CRISPR/Cas9 Mediated Gene Knockdown System in 293T Cells

The lentiCRISPR-v2 vector was received as a gift from Feng Zhang (Addgene plasmid #52961). Feng Zhang’s laboratory online program^1^ was used in designing the sgRNA targets. The two sgRNA target sequences of PRMT5-CRISPR (GAATTGC GTCCCCGAAATAG & CCCGCGTTTCAAGAGGGAGT) used in the study were directed to exon 1 and 2, respectively, of the *Prmt5 gene.* The other two sgRNAs targeted *EGFP* gene (GGG CGAGGAGCTGTTCACCG & GAGCTGGACGGCGACGTA AA) and these were used as controls. Both the *Prmt5 and EGFP* sgRNAs were cloned into the same vector backbone. Cloning was performed according to the Addgene guidelines and as previously described (Shalem et al., 2014; Scaglione et al., 2018). The lenti-CRISPR viruses were generated by transfecting 293T cells with CRISPR/Cas9 plasmids and packing plasmids, psPAX2 and pMD2.G (from Addgene #12260 and #12259). For transfection of every 10-cm dish of 293T cells with polyethylenimine, 10 μg of lenti-CRISPR/Cas9 plasmids, 6 μg of psPAX2 and 2 μg of pMD2.G plasmids were used. The 293T cells were cultured in 293T medium (DMEM, 1mM sodium pyruvate, 2 mM L-glutamine) supplemented with 10% FBS. Tissue culture media were refreshed 15 h post transfection and media containing viruses were harvested 45 h post transfection. Lenti-X^TM^ concentrator kit (Clontech) was used in concentrating viruses.

### Prmt5 Knockdown in Immortalized Oligodendrocyte Progenitors

Immortalized oligodendrocyte progenitors (0.5 million cells) were split into each 10-cm dish 24 h prior to viral infection. Concentrated virus was added into the tissue culture medium of the cells to be infected. The culture medium was supplemented with 4 μg/ml of polybrene. Virus-containing media were replaced by fresh media 8 h post infection. To select for transfected cells, 1 μg/ml of puromycin was added to the medium 2 days post transfection. For experimental analysis, the infected cells were harvested 6 days after infection.

### Western Blotting

Western blotting was done by sodium dodecyl sulfate–polyacrylamide gel electrophoresis (SDS-PAGE) followed by a wet transfer of the proteins onto a polyvinylidene fluoride (PVDF) membrane. The membranes were blocked for 1 h in 10% milk/0.1% Tween/TBS. Primary antibodies were incubated overnight at 4°C in 5% milk/0.1% Tween/TBS. Primary antibodies used include: rabbit-PRMT5 (Abcam ab109451, 1:5000), mouse-GAPDH (Abcam ab8245, 1:5000), total H3 rabbit-(Abcam ab1791, 1:200000), rabbit anti-symmetric Di-Methyl Arginine Motif (Cell Signaling 13222S, 1:1000) and rabbit anti-asymmetric Di-Methyl Arginine Motif (Cell Signaling 13522S, 1:1000). Membranes were washed with 0.1% Tween/TBS and incubated at room temperature for 1 h with horseradish peroxidase conjugated secondary antibodies (Jackson Immunoresearch, 1:10,000) 5% milk/0.1% Tween/TBS. ECL Prime Wester Blotting Detection Reagent kit (GE Healthcare, RPN2232) were then used to develop the membrane. Ponceau staining was performed with Ponceau S solution (Sigma) according to the manufacturer’s instruction. Image J was used to quantify the protein bands.

### Subcellular Protein Fractionation

To sperate cellular extracts into cytosolic and nuclear fractions, cell pellets were treated with hypotonic buffer (10 mM HEPES, pH 7.9, 1.5 mM MgCl_2_, 10 mM KCl) supplemented with 0.5 mM DTT, 1 mM phenylmethylsulfonyl fluoride (PMSF), and protease inhibitor cocktail. After incubation of the re-suspended cells (by rotation at 4°C for 15 min), the membranes of the cells were disrupted by the addition of 0.5% NP40 and vortexed for 10 sec. Cell lysates were centrifuged at 1,500 × *g* for 10 min at 4°C to separate cytoplasmic components (supernatant) from nuclei-enriched fractions (pellets). Cytoplasmic extraction buffer (10×) (0.3 M HEPES, 1.4 M KCl and 30 mM MgCl_2_) was added to the supernatant and sonicated for 5 min (30 s ON/OFF) at high power in the Bioruptor (Diagenode). The soluble fraction was collected as cytoplasmic extract after centrifugation at 16,000 × *g* for 10 min at 4°C. The nuclei-enriched pellet initially obtained was then washed twice with hypotonic buffer + 0.5% NP40. The washed nuclei pellets were re-suspended in mild salt buffer (20 mM HEPES, pH 7.9, 10% glycerol, 1.5 mM MgCl_2_, 0.2mM EDTA and 150 mM KCl) supplemented with 0.5 mM PMSF and protease inhibitor cocktail. The re-suspended pellets were rotated in 4°C for 20 min. The solution was centrifuged at 10,000 × *g* for 10 min at 4°C. The soluble fraction was collected as soluble nuclear protein extract.

### Immunoprecipitation

Primary oligodendrocyte progenitors kept either in the presence or absence of mitogens supplemented with T3 for 48 h (four preparations) were lysed in NP-40 lysis buffer (50 mM HEPES, pH 7.4, 150 mM NaCl, 10% glycerol, 1 mM EDTA, pH 8 and 1% NP-40) supplemented with protease inhibitor cocktail, PMSF, phosphatase inhibitor and TSA. The re-suspended cells were then incubated and rotated at 4°C for 2 h followed by light sonication at low power for 5 min (30 s ON/OFF). After sonication, the samples were centrifuged at 15,000 × *g* for 10 min at 4°C and the supernatant saved. Protein concentration was measured and diluted (if needed) into 1–2 mg/ml with lysis buffer. For immunoprecipitation, 5 mg of total proteins for each IP reaction were used. The lysates were pre-cleared with 10 μg of normal IgG for 2 h at 4°C, followed by addition of 50 μL of protein-A. Each sample was divided into two aliquots. One aliquot was immunoprecipitated with 10 μg of PRMT5 antibody (Abcam, ab109451) while 10 μg of normal IgG were added to the other aliquot and served as control. The samples were left in rotation at 4°C overnight. Next, 50 μl of protein-A beads were added into each sample and left in rotation for 2 h at 4°C. Following centrifugation at 3,000 × *g* for 30 s at 4°C, the immunoprecipitated complexes were washed with 500 μl of lysis buffer supplemented with the inhibitors for a total of five times. Elution was achieved by boiling the samples at 95°C for 10 min in 50 μl of 5× WB loading buffer supplemented with beta-mercaptoethanol. Upon rapid cooling of the samples on ice followed by centrifugation at 15,000 × *g* for 2 min, the supernatant was used for in gel tryptic digestion and LC-MS/MS.

### In Gel Tryptic Digestion and Liquid Chromatography-Tandem Mass Spectrometry for Interactome Samples

The immunoprecipitated samples were run ∼1 cm into SDS-PAGE (Invitrogen NuPAGE Bis-Tris 1.5 mm 10% gel) and stained with Coomassie R250. Gel plugs were subjected to in-gel reduction, alkylation, tryptic digestion, and peptide extraction as described in Shevchenko et al. (1996) and Sleat et al. (2008). Resulting peptides were analyzed by nano LC-MS/MS (Dionex Ultimate 3000 RLSCnano System interfaced with a Velos-LTQ-Orbitrap (Thermo Fisher Scientific, San Jose, CA, United States) as described in (Sleat et al., 2013).

### Interactome Data Analysis

Proteome Discoverer was used to generate mgf files and data were searched against the Ensembl mouse database (Mus_musculus.GRCm37.pep.all.fasta) using an in house version of X!Tandem (GPM cyclone, Beavis Informatics Ltd, Winnipeg, Canada) (Beavis, 2006). Precursor ion mass error tolerance was set to ± 10 ppm and fragment mass error tolerance to ± 0.4 Da. Cysteine carbamidomethylation was set as a complete modification. Acetylation on protein N-termini and oxidation on methionine were set as variable modifications during the first pass search. Deamination of glutamine and asparagine, dioxidation on methionine, oxidation, and dioxidation on tryptophan were set as variable modifications during the search refinement. Trypsin was set for protein cleavage with one or five missed cuts allowed during the initial and refinement searches, respectively. Peptide-spectrum matches with an expectation score of 0.01 were included in the final report. The peptides were grouped into proteins using strict parsimony principle. The spectral counts were analyzed as previously described (Qian et al., 2008). Only proteins with at least two unique peptides detected in the samples immunoprecipitated with PRMT5 antibody, and not in the IgG group, were considered for further analysis. Protein names were converted into gene names and gene ontology analysis was performed using DAVID software. To determine the most abundant molecular functions driven by PRMT5 interactors, we considered categories including at least 1% of the total protein interactors. The percentages were calculated by using only unique proteins in each of the molecular functional categories.

### Protein Digestion, Isobaric Tags for Relative and Absolute Quantitation Labeling, and LC-MS/MS

The protein samples included cytosolic and nuclear fractions, which were independently processed and 0.7 mg/sample for cytosolic fractions and 0.9 mg/sample for nuclear fractions were digested with trypsin, labeled with the iTRAQ reagents following manufacturer’s instruction (ABSciex). iTRAQ samples from each genotype were pooled together and a small fraction (5%) was initially analyzed by quantitative nanoLC-MS/MS, thereby verifying that similar protein amounts were present in both samples. Total reporter ion intensities for each iTRAQ label were determined to be in a similar range in both samples, further validating the similar protein amounts and used as normalization factors in analysis of enriched methylated peptides. This served as control for minor variations in amounts of protein analyzed along with digestion and labeling efficiency. The rest of the samples from each fraction from PRMT5 silenced cells and controls, were subjected to an affinity enrichment using an immobilized antibody recognizing symmetric dimethyl arginine residues (Cell Signaling, 13563) following manufacturer’s standard protocol. At the elution stage, the beads were eluted with 0.15% TFA (trifluroracetic acid) and desalted/concentrated using STAGE trips (Rappsilber et al., 2007). Each of these eluted fractions were analyzed by quantitative LC-MS/MS using a Dionex Ultimate 3000 RLSCnano System interfaced with a QExactive HF (Thermo Fisher Scientific, San Jose, CA) with parameters described in Sleat et al. (2019). The iTRAQ data analysis and statistics were performed using an in-house program described in Sleat et al. (2017). Ontology analysis of the identified non-histone substrates of PRMT5 was performed after converting protein names to gene names using DAVID software. To exclude the inaccurate attribution of substrates due to the effects of PRMT5 knockdown on transcription, we excluded from all the identified substrates those which overlapped with the downregulated transcript list from Scaglione et al. (2018).

### Data Availability

Proteomics MS data (interactome and iTRAQ) have been deposited at Mass Spectrometry Interactive Virtual Environment (MassIVE) with accession number MSV000088396. They can be accessed with the following URL: https://massive.ucsd.edu/ProteoSAFe/static/massive.jsp.

## Results

### Identification of Novel Interactors of PRMT5 in Oligodendrocyte Lineage Cells

**FIGURE 1:**
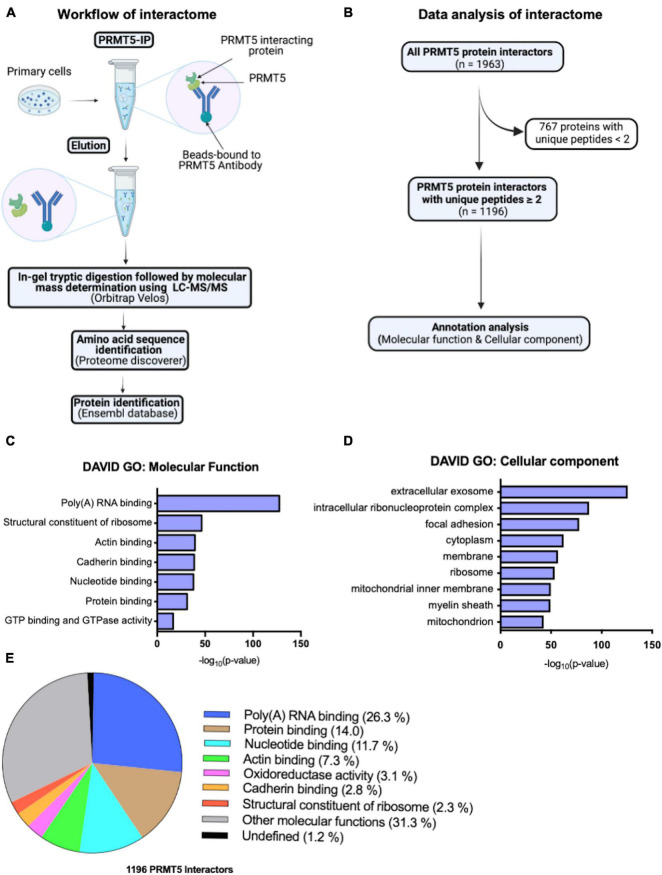
PRMT5 interacting partners in oligodendrocyte lineage cells. **(A)** Schematic diagram of PRMT5 immunoprecipitation (IP) of primary oligodendrocyte lineage cell extracts, followed by mass spectrometry analysis to identifying the interacting partners of PRMT5. **(B)** Summary of the steps involved in the analysis of the interactome data. **(C,D)** Molecular functions **(C)** and cellular localizations **(D)** of PRMT5 interactors in oligodendrocyte lineage cells. Gene ontology analysis was performed using DAVID software. **(E)** The pie-chart shows the proportions of unique proteins involved in the selected molecular functions.

To start identifying potential novel interacting proteins of PRMT5 in oligodendrocyte lineage cells we performed an exploratory analysis using LC-MS/MS. Briefly, peptides were obtained by tryptic digestion of eluates from protein lysates that were immunoprecipitated either with a PRMT5 specific antibody or with a non-specific IgG as control (Figure 1A). The putative PRMT5 interacting proteins were identified using Ensembl, by analyzing those peptides with at least two counts in the PRMT5 immunoprecipitates but not in the IgG control (Supplementary Table 1). After removal of contaminants, the remaining 1196 murine proteins were referred to their relative gene names and further analyzed, using DAVID Gene Ontology analysis to identify functional categories (Figure 1B). As internal validation for the analysis, we retrieved several known interactors of PRMT5. Of note, we detected the methylosome protein 50 (MEP50, encoded by *Mep50/Wdr77*), a molecule that forms a complex with PRMT5 and correctly positions substrates for increased binding affinity (Antonysamy et al., 2012; Burgos et al., 2015). Other known PRMT5 interactors identified by our analysis include: the Band 4.1-like protein 3 (E41L3, encoded by *Epb41l3*) (Jiang et al., 2005), the small nuclear ribonucleoprotein Sm D1 (SMD1, encoded by *Snrpd1*) (Meister et al., 2001; Pesiridis et al., 2009), the TNF receptor-associated factor 4 (TRAF4, encoded by *Traf4*) (Rozan and El-Deiry, 2006; Yang F. et al., 2015), and the zinc-finger protein 326 (ZN326, encoded by *Znf326)* (Rengasamy et al., 2017). In addition, gene ontology analysis of molecular function revealed an enrichment of proteins involved in poly(A)RNA binding, ribosomal structure and nucleotide binding, but also in categories related to cadherin, actin binding and GTPase activity (Figure 1C and Supplementary Table 2). Gene ontology analysis of cellular localization identified mostly a function of PRMT5 in the extracellular exosomes, cytosol, and also ribosome and membrane organelles (Figure 1D and Supplementary Table 2). Consistent with previous reports in other cell types (Rengasamy et al., 2017; Radzisheuskaya et al., 2019), the largest proportion of the PRMT5 protein interactors was involved in molecular functions related to RNA binding (26.3%), protein binding (14.0%) and nucleotide binding (11.7%), with a minor proportion related to actin (7.3%) or cadherin (2.8%) binding and 31.3% related to other molecular functions (Figure 1E). Together, these results suggest that the most prominent function of PRMT5 is conserved in several cell types and is related to RNA processing, although other functions related to actin-dependent events and focal adhesion or cell-cell contacts were also identified in the OPC.

### Identification of PRMT5 Non-histone Substrates in Immortalized Oligodendrocyte Progenitors

**FIGURE 2:**
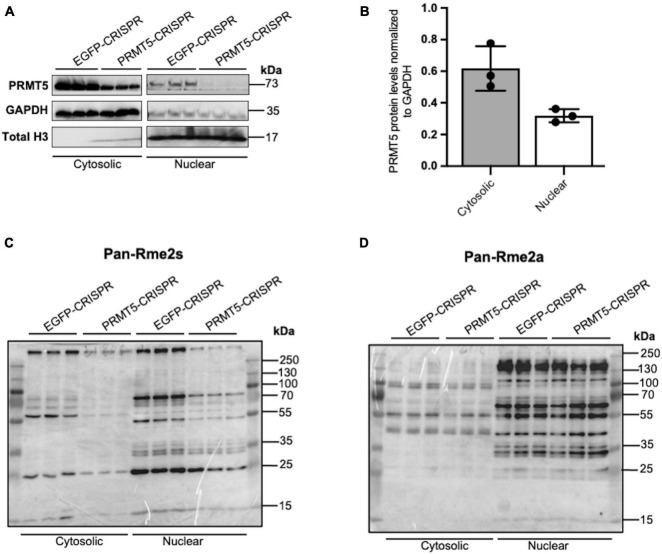
Knockdown of PRMT5 expression using lentiviral CRISPR/Cas9 decreased symmetric but not asymmetric arginine dimethylation in immortalized progenitors. **(A)** Western blots documenting the levels of PRMT5 protein in cytosolic and nuclear protein extracts from immortalized progenitors infected with PRMT5-CRISPR lentiviruses or EGFP-CRISPR as control. **(B)** Quantification of PRMT5 protein levels in cytosolic and nuclear fractions of EGFP-CRISPR control and PRMT5-CRISPR cells. PRMT5 levels in both the cytosolic and nuclear fractions were normalized to GAPDH **(C,D)** Western blot performed with the same protein extracts as **(A)** but probed with antibodies specific for symmetrically dimethylated arginine residues (Rme2s) **(C)** and asymmetrically dimethylated arginine residues (Rme2a) **(D)**.

**FIGURE 3:**
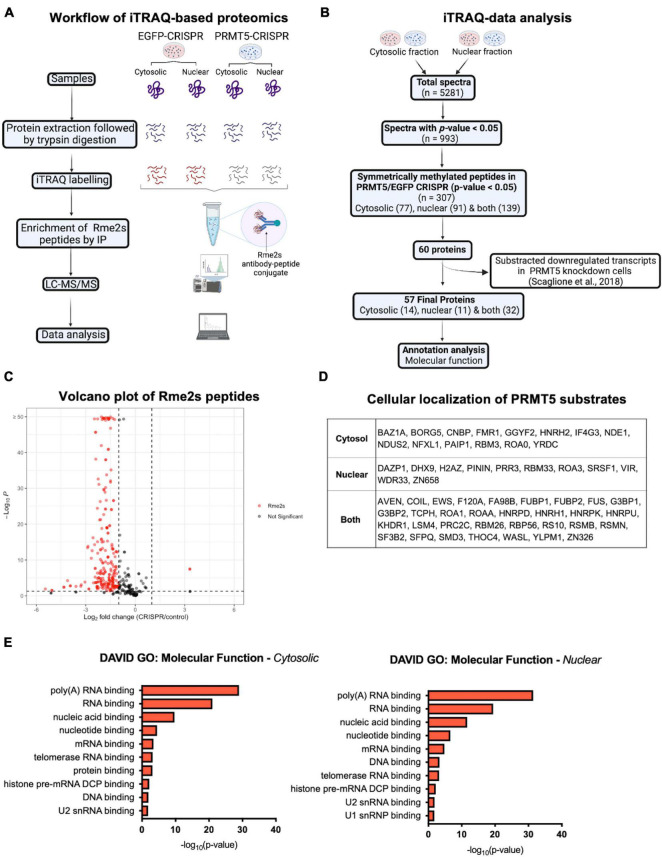
iTRAQ-based proteomics analysis reveals potential substrates of PRMT5 in immortalized progenitors. **(A)** Schematic diagram of the experimental design and workflow of iTRAQ labeling and LC-MS/MS analysis of cytosolic and nuclear extracts from PRMT5 knockdown and EFGP CRISPR controls. **(B)** Flow chart of data analysis after iTRAQ-based proteomic. **(C)** Volcano plot of the 307 immunoaffinity enriched peptides with symmetrically dimethylated arginine residues in control cells, relative to PRMT5-CRISPR cells. On the *X* axis are values of Log_2_ (PRMT5-KD/control) and on Y axis are the -log10 (*p*-values). The red dots represent peptides with fold change < -1 or > 1 and with *p*-value < 0.05. The gray dots represent peptides outside these intervals and level of significance. The plot was generated by combining all the peptides detected in nuclear and cytoplasmic fractions. **(D)** List of the PRMT5 substrates identified after analysis of the iTRAQ datasets generated with nuclear and cytosolic extracts from oligodendrocyte lineage cells. **(E)** Molecular functions of PRMT5 non-histone substrates identified in the cytosolic (left) and nuclear (right) fractions. Gene ontology analysis was performed using DAVID software.

In order to gain further insights on PRMT5 substrates in the oligodendrocyte lineage, we then used isobaric Tags for Relative and Absolute Quantitation (iTRAQ) of cytosolic or nuclear extracts from immortalized oligodendrocyte progenitors either silenced with lentiviral-mediated PRMT5 CRISPR-Cas9 (PRMT5-CRISPR) or transduced with EGFP-CRISPR, as control. The efficiency of the knockdown was validated using western blot analysis (Figure 2A). Decreased levels of PRMT5 in both cytosolic and nuclear extracts were detected in PRMT5-CRISPR cells compared to EGFP-CRISPR controls, although the total number of proteins was shown to be comparable (see section “Materials and Methods”) in PRMT5 knockdown cells and controls (Figure 2B). As expected, PRMT5 enzymatic activity was decreased in the cytosolic and nuclear extracts from PRMT5 knockdown cells compared to EGFP-CRISPR controls, as indicated by Western-blot analysis, using an antibody recognizing all the proteins with symmetrically dimethylated arginine residues (Figure 2C). Multiple immunoreactive bands across a wide range of molecular weights (15–250 kDa) were detected in the EGFP-CRISPR controls, while their signal intensity was either lost or reduced in PRMT5-CRISPR samples (Figure 2C), suggesting the existence of several proteins with decreased levels of symmetrically methylated arginine residues in knockdown cells. Importantly, the reduction or absence of proteins with symmetric methylated arginines was not met by increased levels of proteins with asymmetric arginine methylation, suggesting that Type I PRMTs did not compensate for decreased Type II PRMT function (Figure 2D). To define the identity of the PRMT5 substrates in immortalized progenitors, we utilized iTRAQ-based mass spectrometry of cytosolic and nuclear extracts (Figure 3A). We digested the cytosolic and nuclear protein samples into peptides and followed by iTRAQ labeling, we then used specific antibodies to enrich for peptides containing symmetric dimethyl arginine residues. Samples were subjected to LC-MS/MS analysis and symmetrically dimethylated arginine levels of each peptide were quantified in cytosolic and nuclear extracts from PRMT5 knockdown cells and controls. Of the peptides with a minimum of three spectral counts in control samples, 307 harbored at least one symmetric dimethyl arginine site (*p*-value < 0.05) (Supplementary Table 3). Of those, 77 peptides were detected as differentially methylated only in the cytosolic fraction, 91 only in the nuclear fraction, and 139 peptides were found in both fractions (Figure 3B). Therefore, a similar number of peptides were found to be differentially methylated in either the cytosolic (*n* = 216) or nuclear fraction (*n* = 230) of the PRMT5 knockdown cells compared to the EGFP controls. These peptides corresponded to 60 putative protein substrates with significantly different arginine methylation in knockdown cells compared to controls. We then subtracted the proteins whose transcript levels were downregulated by PRMT5 knockdown [as we published in Scaglione et al. (2018)], in order to avoid the potential confounder of the transcriptional effect of PRMT5. The new analysis identified 57 substrates (Figure 3B). All the 307 peptides identified in both fractions, with their respective fold changes in knockdown cells and compared to controls and relative *p-*values are shown in the volcano plot (Figure 3C and Supplementary Table 3). The identified 57 substrates with 14 proteins detected only in the cytosolic fraction, 11 proteins only in the nuclear fraction and 32 in both (Figure 3D). These data do not suggest a preferential methylation of substrate in one fraction versus the other. Substrates were characterized by the presence of symmetrically methylated arginine residues flanked by glycine residues, identifying the RGG or RG motif as previously reported (Thandapani et al., 2013 and Supplementary Table 4), with very few exceptions. PRMT5 substrates identified only in the cytosolic fraction of oligodendrocyte lineage cells included: the zinc-finger binding protein CNBP (encoded by *Cnbp*), the temperature responsive mRNA binding protein RBM3 (encoded by *Rbm3*), the mRNA trafficking protein FMR1 (encoded by *Fmr1*), the chromatin remodeling complex BAZ1A (Encoded by *Baz1a)* and the repressor of translation initiation GGYF2 (encoded by *Gigyf2)*. Substrates identified in the nuclear fraction included: the multi-functional ATP binding helicase DHX9 (encoded by *Dhx9)* expressed at high levels in the oligodendrocyte lineage and involved in the positive regulation of nuclear export of constitutive transport element (CTE)-containing unspliced mRNA (Lee and Pelletier, 2016), the histone protein H2Az (encoded by *H2az1)*, and the transcription factor ZN658 (encoded by *Zfp658*). Substrates identified in both cytosolic and nuclear fractions included: proteins regulating pre-mRNA splicing (e.g., RSMB encoded by *Snrpb*, SMD3 encoded by *Snrpd3*), brain-specific RNA splicing (e.g., RSMN encoded by *Snrpn*), regulation or packaging of pre-mRNA into hnRNP particles (e.g., HNRH1 encoded by *Hnrnph1*) and transport from the nucleus to the cytoplasm (e.g., ROA1 encoded by *Hnrnpa1*), RNA decay and mRNA turnover (e.g., HNRPD encoded by *Hnrnpd*) as well as the methylated RNA binding proline-rich protein PRC2C (encoded by *Prrc2c)* (Figure 3D). Next, we performed gene ontology with the unique substrates detected in the cytosolic or nuclear fraction using DAVID Ontology software (Figure 3E and Supplementary Table 5). Regardless of the fraction where the proteins were identified, PRMT5 substrates were overwhelmingly represented in categories such as mRNA processing, RNA splicing, regulation of mRNA stability and positive regulation of translation, with the nuclear fraction additionally showing histones and DNA binding proteins. Worth noticing is the fact that some nuclear proteins were found to be methylated in the cytoplasmic fraction, possibly suggesting a role for symmetric arginine methylation in regulating nuclear/cytosolic shuttling and nuclear transport.

### Comparative Analysis of PRMT5 Substrates in Immortalized Progenitor Cells and Other Cell Lines

**FIGURE 4:**
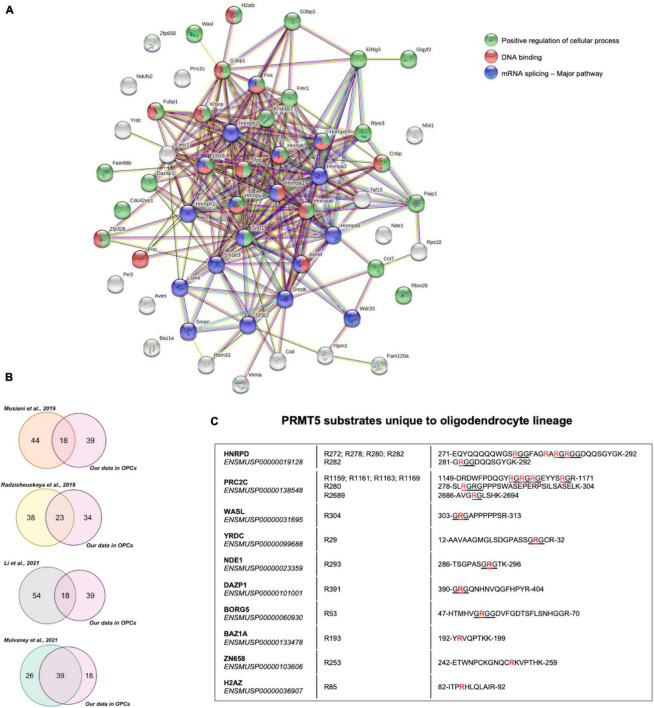
Comparative analysis of PRMT5 protein substrates. **(A)** Functional connections among the 57 PRMT5 substrates identified in oligodendrocyte lineage cells using the STRING protein-protein interaction database (http://string-db.org/), with Medium Confidence (0.400) threshold parameter. Proteins involved in a common pathway are indicated by same-colored nodes (top right). Lines between nodes represent distinct associations between proteins, with line colors indicating different types of relationships: neighborhood (green), gene fusion (red), co-occurrence (dark blue), co-expression (black), experimental evidence (pink), database (turquoise), text mining (light green), and homology (violet). **(B)** Venn diagrams representing the overlaps of PRMT5 substrates between those reported in the indicated studies and the ones identified in our current study. **(C)** List of PRMT5 substrates that are unique to the oligodendrocyte lineage cells. The table shows the detected substrates, their Ensembl IDs, the specific symmetrically methylated arginine residues within the peptide sequences of each substrate. The symmetrically dimethylated arginine residues are in red and the canonical RGG/RG motifs recognized by PRMT5 are underlined.

Functional interactions among the 57 PRMT5 substrates were analyzed using Search Tool for the Retrieval of Interacting Genes/Proteins (STRING) and validated the importance of PRMT5 substrates in regulating three main processes: overwhelmingly mRNA splicing and related pathways, but also DNA binding and other cellular processes involved in migration and other functions (Figure 4A). We then compared the PRMT5 substrates identified by our study in oligodendrocyte lineage cells to those previously reported in distinct tumors and cell lines (Supplementary Table 6). Of the 57 substrates, 18 proteins (31.6%) were in common with the substrates previously identified in HeLa cells (Musiani et al., 2019), 23 proteins (40.3%) were in common with those identified in human AML cells (human acute myeloid leukemia) (Radzisheuskaya et al., 2019), 18 proteins (31.6%) in common with HEK293T cells (Li et al., 2021), and 39 proteins (68.4%) with the MiaPaca2 cells (human pancreatic cancer cell line) (Mulvaney et al., 2021; Figure 4B). Among the proteins shared between oligodendrocyte progenitors and the cell lines, we identified the zinc-finger protein ZN326, the RNA binding protein involved in mRNA transport ROA1, and the spliceosome component RSMB, suggesting a common role of PRMT5 in related processes in distinct cell types (Supplementary Table 6). Ten PRMT5 substrates were identified only in the oligodendrocyte lineage cells and included HNRPD, a molecule involved in RNA stability and circadian regulation of translation (Fialcowitz et al., 2005; Kojima et al., 2011), the cytoplasmic proline-rich PRC2C substrate, with the ability to bind methylated RNA (Wu et al., 2019), the WASP Like Actin Nucleation Promoting Factor WASL, expressed at high levels in neural tissue (Fukuoka et al., 1997), the nuclear NudE Neurodevelopment protein 1 NDE1, which is involved in microtubule organization and neuronal migration (Feng and Walsh, 2004; Sasaki et al., 2005) and the RNA binding protein DAZP1. Among the oligodendrocyte unique substrates, with atypical PRMT5 methylation sites we also identified the zinc-dependent transcriptional repressor ZN658, the histone H2Az previously reported to be involved in gliogenesis (Su et al., 2018) and the component of the ATP-dependent chromatin remodeling complex BAZ1A, which is enriched in the newly formed oligodendrocytes (Sharma et al., 2015; Zeisel et al., 2015).

## Discussion

Arginine methylation is an energetically demanding modification, requiring the use of 12 ATP molecules for each methylation event (Gary and Clarke, 1998) resulting in a change of the shape of arginine residues, which lose their ability to bind to hydrogen bond acceptor proteins (Fuhrmann et al., 2015), while gaining the ability to bind to other proteins (Hughes and Waters, 2006). Methylation is catalyzed by the PRMT family of enzymes, with asymmetric methylation of RxR motifs catalyzed by class I PRMTs (e.g., PRMT1) and symmetric methylation of RGG/RG motifs catalyzed by class II (e.g., PRMT5). Intriguingly PRMT1 deletion in neural stem cells significantly reduced the ability of oligodendrocyte progenitor cells to differentiate into myelin-forming cells while it had no effect on astrocytic and neuronal differentiation (Hashimoto et al., 2016). Oligodendrocyte lineage specific deletion of *Prmt5* impacted survival of newly formed oligodendrocytes and impaired differentiation (Scaglione et al., 2018), thereby suggesting a critical role of PRMT5 in this lineage. PRMT1 has been shown to mostly function as a transcriptional activator by recruiting histone acetyltransferase to the asymmetrically methylated R3 residue in histone H4 (H4R3me2a) and facilitating acetylation of several lysine residues (Huang et al., 2005), while PRMT5 has been characterized as mediating transcriptional repression by competition of H4R3me2s with K5 residue acetylation (Scaglione et al., 2018). However, only a small fraction of the PRMT5 protein displayed nuclear localization in the oligodendrocyte lineage cells and spurred the interest to identify the existence of cell-specific interactors and substrates. The analysis presented in this study provides an initial description of the proteins specifically immunoprecipitated by a PRMT5-specific antibody but not by a non-specific IgG. Of the several interactors, we identified previously reported protein partners (Liang et al., 2021), such as WDR77/MEP50 (Antonysamy et al., 2012; Burgos et al., 2015) and several other molecules reported in other cell types, such as Small nuclear ribonucleoprotein Sm D1 (Meister et al., 2001; Pesiridis et al., 2009), TNF receptor-associated factor 4 (Rozan and El-Deiry, 2006; Yang F. et al., 2015) and transcription factor ZN326 (encoded by *Znf326)* (Rengasamy et al., 2017). At odds with previous reports on the role of PRMT5 in regulating the cell cycle, either by affecting histone methylation (F. Liu et al., 2020) or by direct methylation of P53 and E2F1 in cancer cells (Pastore et al., 2020), we did not detect any cell cycle regulatory molecule in our study. These results are also consistent with the lack of a proliferation-related phenotype in mice with oligodendrocyte lineage specific *Prmt5* knockout (Scaglione et al., 2018) and further reinforce the concept that the physiological role of PRMT5 is distinct from that studied in cancer cells.

We then searched for PRMT5 substrates using iTRAQ analysis of cytosolic and nuclear protein extracts from oligodendrocyte lineage cells with PRMT5 knocked down compared to control cells, immunoprecipitated with antibodies recognizing symmetrically methylated arginine residues. We only analyzed peptides with symmetrically methylated arginine residues in control, but not in PRMT5 knockdown cells. We then obtained the proteins corresponding to those peptides and eliminated from the results any molecule whose transcripts we had previously reported to be downregulated in Prmt5 knockdown cells (Scaglione et al., 2018). This left us with the identification of 57 protein substrates identified in the nuclear and cytosolic extracts. In common with many cell lines (Musiani et al., 2019; Radzisheuskaya et al., 2019; Li et al., 2021; Mulvaney et al., 2021), we detected the majority of PRMT5 substrates involved in RNA splicing and processing. Among the common PRMT5 substrates (Musiani et al., 2019; Radzisheuskaya et al., 2019; Li et al., 2021; Mulvaney et al., 2021), we identified: ROA1, a protein regulating translation at the internal ribosomal entry site (Gao et al., 2017), the ribosomal protein RS10, whose arginine methylation is essential for proper ribosome assembly, and the zinc-finger protein ZN326, enriched in the oligodendrocyte lineage (Zhang et al., 2014; Sharma et al., 2015) and regulated throughout development, with the highest expression in neuronal tissues in E11.5 embryos (Lee et al., 2000). ZN326 complexes with nuclear messenger ribonucleoproteins (mRNPs) and Deleted in Breast Cancer 1 (DBC1) to form the regulatory DBIRD complex. The DBIRD complex binds to RNA polymerase II (RNAPII) and regulates alternative splicing (exon skipping) by facilitating transcriptional elongation at junctions between introns and AT rich exons (Close et al., 2012; Rengasamy et al., 2017). By symmetrically methylating ZN326, PRMT5 ensures the exclusion of AT rich exons as their inclusion has profound destabilizing effects on RNA (Close et al., 2012; Rengasamy et al., 2017). ZN326 has also been reported to positively regulate the WNT pathway and HDAC transcription activities in glioma (Yu et al., 2019), both implicated in timing of oligodendrocyte development in the central nervous system (Marin-Husstege et al., 2002; Conway et al., 2012).

Among the protein substrates uniquely identified in the oligodendrocyte lineage cells we found several proteins enriched in neural tissue, such as chromatin remodeling protein BAZ1A, the ATP-dependent helicase DHX9 and several molecules regulating the microtubule and actin cytoskeleton, including WASL, BORG5, and NDE1. We also detected HNRPD, a component of a family of proteins regulating the maturation of the heterogeneous nuclear RNAs (hnRNAs) into messenger RNAs (mRNA) and their subsequent translation (Geuens et al., 2016). Due to its affinity of for AU-rich mRNA-destabilizing sequence in the 3′-UTR region of mRNA, HNRPD is thought to regulate mRNA decay (Fialcowitz et al., 2005), such as the half-life of immediate-early genes and the circadian regulation of circadian rhythm genes, such as *Cry* and *Per* (Kojima et al., 2011).

PRC2C, an RNA-binding protein seen in few studies (Baltz et al., 2012; Castello et al., 2012, 2016), is an additional PRMT5 substrate that is unique to oligodendrocyte lineage cells. In a recent study in neural cells, PRC2C and PRC2A were both identified as novel readers of N^6^-methyladenosine (m^6^A), a methylated adenosine modification in mRNA (Wu et al., 2019). While the role of PRC2C protein in oligodendrocyte lineage cells is yet to be investigated, a closely related protein, PRC2A has been shown to control oligodendroglial specification and myelination (Wu et al., 2019).

In summary, our data suggest a critical role of PRMT5 in regulating mRNA metabolism, but also transcription and translation. A potential limitation of our analysis is the focus on PRMT5 substrates in the cytosolic and nuclear fractions of oligodendrocyte lineage cells, thereby leaving out substrates potentially enriched in other subcellular compartments (such as the plasma membranes or internal organelles). A notable example is the membrane associated PDGFRα, previously identified as PRMT5 target in OPCs (Calabretta et al., 2018). Overall, future studies will be needed to fully elucidate the role of arginine methylation in brain health and in disease states.

## Data Availability Statement

The datasets presented in this study can be found in online repositories. The names of the repository/repositories and accession number(s) can be found below: CCMS MassIVE, accession no: MSV000088396.

## Ethics Statement

This animal study was reviewed and approved by IACUC at Icahn School of Medicine.

## Author Contributions

DD analyzed the data relative to interacting partners and substrates, prepared the figures, wrote the first draft of the text, and edited the manuscript with PC. JL prepared the cell extracts and worked with PC and HZ in experimental design. IS performed the substrate data analysis and comparative analysis with other studies and helped with figure preparation and text editing. HZ and DM helped with the design and performance of the iTRAQ experiment and mass spectrometry, analyzed the raw data, and contributed to manuscript editing. PC worked with JL on the initial experimental design and conceptualization, wrote the text with DD, and supervised the overall workflow. All authors contributed to the article and approved the submitted version.

## Conflict of Interest

The authors declare that the research was conducted in the absence of any commercial or financial relationships that could be construed as a potential conflict of interest.

## Publisher’s Note

All claims expressed in this article are solely those of the authors and do not necessarily represent those of their affiliated organizations, or those of the publisher, the editors and the reviewers. Any product that may be evaluated in this article, or claim that may be made by its manufacturer, is not guaranteed or endorsed by the publisher.

## Footnotes

1
http://crispr.mit.edu/

## Supplementary Material

Supplementary Table 1 Interacting partners of PRMT5 in oligodendrocyte lineage cells. List of PRMT5 interactors in oligodendrocyte lineage cells identified by their gene symbols and ranked according to the number of identified unique peptides.

Supplementary Table 2 Ontology terms of PRMT5 interacting partners in oligodendrocyte lineage cells. Top 50 molecular functions of PRMT5 interactors in oligodendrocyte lineage cells. Gene ontology was performed using DAVID analysis. The GO terms and relative *p*-values are shown.

Supplementary Table 3 Peptides (*n* = 307) with differential Rme2s in PRMT5 KD cells compared to EGFP controls. The first column shows the total number of peptides with symmetrically methylated arginine residues that were identified in the control cells as significantly different from the PRMT5 knockdown cells. The corresponding log2foldchange and *p*-values of the significant peptides are shown in the second and third column, respectively.

Supplementary Table 4 Symmetrically dimethylated arginine residues of PRMT5 substrates in oligodendrocyte lineage cells. List of PRMT5 substrates, their Uniprot Protein Identification Codes and accession numbers, and the specific arginines residues identified as symmetrically dimethylated in the controls but not in PRMT5 knockdown cells.

Supplementary Table 5 Ontology terms of PRMT5 substrates in oligodendrocyte lineage cells. Molecular functions of PRMT5 substrates identified in cytosolic and nuclear extracts from oligodendrocyte lineage cells. Gene ontology was performed using DAVID analysis. The GO terms and relative *p*-values are shown.

Supplementary Table 6 PRMT5 substrates in different cell lines. List of PRMT5 substrates identified in our study (immortalized oligodendrocyte progenitors), MiaPaca2 cells (Mulvaney et al., 2021), HeLa cells (Musiani et al., 2019), AML cells (Radzisheuskaya et al., 2019), and HEK293 cells (Li et al., 2021). Highlighted in red are substrates whose transcript levels are downregulated in PRMT5 KD cells (Scaglione et al., 2018).
