# Supplementary material for: PRMT5 Interacting Partners and Substrates in Oligodendrocyte Lineage Cells
Source: Front Cell Neurosci. 2022 Mar 17;16:820226. doi: 10.3389/fncel.2022.820226 (PMC8968030; doi:10.3389/fncel.2022.820226)
Supplement: Supplementary Table 1 — Interacting partners of PRMT5 in oligodendrocyte lineage cells. List of PRMT5 interactors in oligodendrocyte lineage cells identified by their gene symbols and ranked according to the number of identified unique peptides. [file Table_1.DOCX]

**Table 1.**

| **Gene symbol** | **Number of unique peptides** |
| --- | --- |
| Sptan1 | 292 |
| Myh10 | 284 |
| Sptbn1 | 263 |
| Myh9 | 239 |
| Plec | 221 |
| Actb | 164 |
| Myh14 | 161 |
| Myo5a | 157 |
| Dync1h1 | 150 |
| Myo18a | 143 |
| Myo6 | 135 |
| Ranbp2 | 127 |
| Tjp1 | 112 |
| Dock9 | 91 |
| Fasn | 89 |
| Cltc | 87 |
| Vim | 81 |
| Actn4 | 81 |
| Tjp2 | 80 |
| Dock7 | 77 |
| Specc1 | 67 |
| Lima1 | 65 |
| Flnb | 63 |
| Itpr2 | 62 |
| Ppp1r9b | 60 |
| Dhx9 | 60 |
| Mprip | 58 |
| Ppp1r12a | 56 |
| Limch1 | 56 |
| Specc1l | 55 |
| Dock10 | 55 |
| Fscn1 | 53 |
| Slc25a5 | 53 |
| Hnrnpu | 50 |
| Dbn1 | 48 |
| Lmnb1 | 48 |
| Sfpq | 47 |
| Hsp90ab1 | 46 |
| Ctnna1 | 46 |
| Epb41l2 | 46 |
| Cnp | 45 |
| Actn1 | 44 |
| Flii | 44 |
| Fmnl2 | 43 |
| Hnrnpm | 42 |
| Pcm1 | 41 |
| Shroom2 | 41 |
| Eef2 | 39 |
| Svil | 39 |
| Ppp1r9a | 39 |
| Tubb2b | 38 |
| Capzb | 38 |
| Ppp1ca | 38 |
| Hspa8 | 38 |
| Ddx5 | 38 |
| Mybbp1a | 37 |
| Snrnp200 | 37 |
| Kif21b | 37 |
| Sptbn2 | 37 |
| Sipa1l1 | 37 |
| Ctnnd2 | 36 |
| Eef1a1 | 35 |
| Arhgap21 | 35 |
| Sipa1l2 | 35 |
| Tuba1a | 34 |
| Hspa5 | 34 |
| Nup153 | 33 |
| Ctnna2 | 33 |
| Prpf8 | 33 |
| Ctnnb1 | 32 |
| Tpm1 | 32 |
| Prmt5 | 32 |
| Myo1b | 32 |
| Immt | 32 |
| Map4k4 | 32 |
| Map1b | 32 |
| Hspa9 | 31 |
| Appl2 | 31 |
| Nup205 | 31 |
| Tpm3 | 30 |
| Ctnnd1 | 30 |
| Macf1 | 30 |
| Atp5a1 | 29 |
| Rpl5 | 29 |
| Hadha | 29 |
| Rps3a1 | 28 |
| Rps4x | 28 |
| Nono | 28 |
| Atp1a2 | 28 |
| Fyn | 28 |
| Nup210 | 28 |
| Arvcf | 28 |
| Smarca4 | 28 |
| Dctn1 | 28 |
| Atp5b | 27 |
| Rps18 | 27 |
| Slc25a12 | 27 |
| Rpl7a | 27 |
| Hspd1 | 27 |
| Copa | 27 |
| Gja1 | 26 |
| Nxf1 | 26 |
| Cntn1 | 26 |
| Pkp4 | 26 |
| Flna | 26 |
| Rps3 | 25 |
| Matr3 | 25 |
| Efhd2 | 25 |
| Capza2 | 24 |
| Slc25a4 | 24 |
| Atp2a2 | 24 |
| Phb2 | 24 |
| Ddx3x | 24 |
| Aif1l | 24 |
| Tom1 | 24 |
| Arpc2 | 24 |
| Rpl8 | 24 |
| Nup155 | 24 |
| Myo1e | 24 |
| Dock4 | 24 |
| Gapdh | 23 |
| Enpp6 | 23 |
| Gnb2 | 23 |
| Hnrnpl | 23 |
| Ncam1 | 23 |
| Vcp | 23 |
| Srcin1 | 23 |
| Aldh6a1 | 23 |
| Rps19 | 22 |
| Rpl10 | 22 |
| Gsn | 22 |
| Rpl6 | 22 |
| Uba1 | 22 |
| Myo1c | 22 |
| Plekha7 | 22 |
| Gnai2 | 21 |
| Rpl7 | 21 |
| Tom1l2 | 21 |
| Ncl | 21 |
| Rpl4 | 21 |
| Sec23ip | 21 |
| C530008M17Rik | 21 |
| Ap2a1 | 21 |
| Pakap | 21 |
| Scrib | 21 |
| Tmod2 | 20 |
| Hist4h4 | 20 |
| Hnrnpa1 | 20 |
| Rps13 | 20 |
| Abcd3 | 20 |
| Coro2b | 20 |
| Hnrnpa3 | 20 |
| Cald1 | 20 |
| Myef2 | 20 |
| Sh3bp4 | 20 |
| Rpn1 | 20 |
| Cyfip2 | 20 |
| Gcn1l1 | 20 |
| Upf1 | 20 |
| Rplp0 | 19 |
| Gcdh | 19 |
| Rpl10a | 19 |
| Vdac1 | 19 |
| Rps6 | 19 |
| Gnb2l1 | 19 |
| Ppp1r18 | 19 |
| Atp5f1 | 19 |
| Ddx17 | 19 |
| Ap2b1 | 19 |
| Dhrs4 | 19 |
| Ddx21 | 19 |
| Atad3a | 19 |
| Capza1 | 18 |
| Srsf1 | 18 |
| Bcas1 | 18 |
| Atp5c1 | 18 |
| Slc25a3 | 18 |
| Twf1 | 18 |
| Kpnb1 | 18 |
| Hnrnpk | 18 |
| Tprn | 18 |
| Coro1c | 18 |
| Rpl3 | 18 |
| Actr3 | 18 |
| Nckap1 | 18 |
| Dst | 18 |
| Sdha | 18 |
| Sf3b1 | 18 |
| Sorbs1 | 18 |
| Gfap | 18 |
| Myo10 | 18 |
| Nes | 18 |
| Chd4 | 18 |
| Acot13 | 17 |
| Rps16 | 17 |
| Elavl1 | 17 |
| Rps7 | 17 |
| Rps5 | 17 |
| Cfl1 | 17 |
| Slc25a18 | 17 |
| Cttn | 17 |
| Rpl27-ps3 | 17 |
| Psmd1 | 17 |
| Frmd4a | 17 |
| Eif3a | 17 |
| Acly | 17 |
| Eprs | 17 |
| Kif5b | 17 |
| Dnm3 | 17 |
| Dlg5 | 17 |
| Myl6 | 16 |
| Hnrnph1 | 16 |
| Rpl13 | 16 |
| Rab1a | 16 |
| Npm1 | 16 |
| Rps10 | 16 |
| Rap1a | 16 |
| Hnrnpa0 | 16 |
| Rps9 | 16 |
| Rpl26 | 16 |
| Cdh2 | 16 |
| Dhx15 | 16 |
| Eif4a1 | 16 |
| Sf3b2 | 16 |
| Srgap2 | 16 |
| Tubb4a | 15 |
| Ckb | 15 |
| Hsd17b12 | 15 |
| Gm10036 | 15 |
| Ppp1cb | 15 |
| Rpl23 | 15 |
| Pabpc1 | 15 |
| Slc25a11 | 15 |
| Rpl28 | 15 |
| Ywhae | 15 |
| Ppap2b | 15 |
| Rab14 | 15 |
| Ppib | 15 |
| Nup93 | 15 |
| Bdh1 | 15 |
| Actr1a | 15 |
| Rpl13a | 15 |
| Snd1 | 15 |
| Trim28 | 15 |
| Epb41 | 15 |
| Psmd2 | 15 |
| Lrch2 | 15 |
| Eftud2 | 15 |
| Tagln3 | 15 |
| Arhgap35 | 15 |
| Elmo1 | 15 |
| Ank2 | 15 |
| Nup98 | 15 |
| Rac1 | 14 |
| Rps8 | 14 |
| Rps2 | 14 |
| Rps17 | 14 |
| Vapa | 14 |
| Rpl18a | 14 |
| Phb | 14 |
| Tollip | 14 |
| Rpl14 | 14 |
| Rpl23a | 14 |
| Rpl9 | 14 |
| Tardbp | 14 |
| Rps11 | 14 |
| Rbm14 | 14 |
| Actr2 | 14 |
| Tmpo | 14 |
| Ralb | 14 |
| Slc25a1 | 14 |
| Nup214 | 14 |
| Ppp1r12b | 14 |
| Hnrnpr | 14 |
| Phldb1 | 14 |
| Hsp90aa1 | 14 |
| Ktn1 | 14 |
| Rps27a | 13 |
| Myl12b | 13 |
| Hnrnpa2b1 | 13 |
| Vdac2 | 13 |
| Rps25 | 13 |
| Rpl17 | 13 |
| Mfge8 | 13 |
| Add1 | 13 |
| Hist1h1c | 13 |
| Actr10 | 13 |
| Lrrc59 | 13 |
| Sfxn3 | 13 |
| Canx | 13 |
| Gtf2i | 13 |
| Mtdh | 13 |
| Ndufs1 | 13 |
| Cad | 13 |
| Prdx1 | 13 |
| Dennd2a | 13 |
| Hmgcs1 | 13 |
| Hip1 | 13 |
| Actc1 | 12 |
| Atp5o | 12 |
| Rps15a | 12 |
| Gnao1 | 12 |
| Rps12-ps3 | 12 |
| Ran | 12 |
| Map2 | 12 |
| Arpc3 | 12 |
| Mbp | 12 |
| Chchd3 | 12 |
| Rrbp1 | 12 |
| Dpysl2 | 12 |
| Traf4 | 12 |
| Gm9755 | 12 |
| Eif3b | 12 |
| Rpl19 | 12 |
| Soga1 | 12 |
| Synpo2 | 12 |
| Cdc42 | 11 |
| Rpl24 | 11 |
| Rhoa | 11 |
| Rpl15 | 11 |
| Kras | 11 |
| Lsamp | 11 |
| Tecr | 11 |
| Ptbp1 | 11 |
| Ywhaz | 11 |
| Hnrnpf | 11 |
| Vdac3 | 11 |
| Gnas | 11 |
| Tns3 | 11 |
| Tpm4 | 11 |
| Ap2a2 | 11 |
| Ppp2r1a | 11 |
| Hsp90b1 | 11 |
| Acsbg1 | 11 |
| Acaca | 11 |
| Arhgef2 | 11 |
| Dapk1 | 11 |
| St5 | 11 |
| Rps14 | 10 |
| Gm9396 | 10 |
| Srsf3 | 10 |
| Rpl21 | 10 |
| Rpl30 | 10 |
| Rab5c | 10 |
| Gnb1 | 10 |
| Rps20 | 10 |
| Rpl22 | 10 |
| Rbmxl1 | 10 |
| Rab6a | 10 |
| Mtch2 | 10 |
| Fabp7 | 10 |
| Ndufs3 | 10 |
| Cyb5r3 | 10 |
| Ndufa9 | 10 |
| Arpc4 | 10 |
| U2af1 | 10 |
| Sfxn1 | 10 |
| Tmod3 | 10 |
| Ywhag | 10 |
| Ywhaq | 10 |
| Eif2s1 | 10 |
| Pycr2 | 10 |
| Nup88 | 10 |
| Acsl3 | 10 |
| Nup160 | 10 |
| Lrrfip2 | 10 |
| Rab10 | 10 |
| Ilf3 | 10 |
| Eps8 | 10 |
| Gfpt1 | 10 |
| Mb21d2 | 10 |
| Dclk2 | 10 |
| Ckap4 | 10 |
| Cct3 | 10 |
| Plxnb3 | 10 |
| Etfa | 10 |
| D8Ertd82e | 10 |
| Add3 | 10 |
| Inppl1 | 10 |
| Sipa1l3 | 10 |
| Myo19 | 10 |
| Arhgap42 | 10 |
| Smc3 | 10 |
| Calm2 | 9 |
| Atp5h | 9 |
| Actbl2 | 9 |
| Tsc22d4 | 9 |
| Rab11b | 9 |
| Cyc1 | 9 |
| Rab7 | 9 |
| Rab31 | 9 |
| Mest | 9 |
| Rps23 | 9 |
| Rpsa | 9 |
| Sf3b3 | 9 |
| Rpl36 | 9 |
| Slc3a2 | 9 |
| Hnrnpul2 | 9 |
| Rpl32 | 9 |
| Hist2h3c2 | 9 |
| Aco2 | 9 |
| Akap5 | 9 |
| Sec22b | 9 |
| Ak1 | 9 |
| Sdhb | 9 |
| Apoe | 9 |
| Hsd17b4 | 9 |
| Dhrs1 | 9 |
| Rpl31 | 9 |
| Rap2a | 9 |
| Tpgs1 | 9 |
| Efhd1 | 9 |
| Ppp2ca | 9 |
| Gnaq | 9 |
| Glipr2 | 9 |
| Atp1a1 | 9 |
| Rcn2 | 9 |
| Gm10123 | 9 |
| Ddx1 | 9 |
| Iqsec1 | 9 |
| Cct2 | 9 |
| Dcakd | 9 |
| Mthfd1 | 9 |
| Nup107 | 9 |
| Mdh2 | 9 |
| Lrch3 | 9 |
| Gipc1 | 9 |
| Aacs | 9 |
| Hist1h2bl | 8 |
| Alyref | 8 |
| Dynll1 | 8 |
| Hist1h2al | 8 |
| Rpl27a | 8 |
| Rab2a | 8 |
| Fus | 8 |
| Hnrnpc | 8 |
| Sfxn5 | 8 |
| Snrpd2 | 8 |
| Rpn2 | 8 |
| Cox4i1 | 8 |
| Wdr77 | 8 |
| Rpl35a | 8 |
| Basp1 | 8 |
| Prdx2 | 8 |
| Hnrnpab | 8 |
| Grtp1 | 8 |
| Pgam5 | 8 |
| Fbl | 8 |
| Eif3c | 8 |
| Sirt2 | 8 |
| Ndufa4 | 8 |
| Hp1bp3 | 8 |
| Copg1 | 8 |
| Dnaja1 | 8 |
| Rdx | 8 |
| Ybx1 | 8 |
| Nfasc | 8 |
| Suclg1 | 8 |
| Dctn2 | 8 |
| Ssfa2 | 8 |
| Rasa3 | 8 |
| Gpam | 8 |
| Lonp2 | 8 |
| Pdhb | 8 |
| Aars | 8 |
| Lrp1 | 8 |
| Smc1a | 8 |
| Slc1a3 | 7 |
| Rps15 | 7 |
| Snrpb | 7 |
| Rps26 | 7 |
| H1fx | 7 |
| Rpl18 | 7 |
| Arf4 | 7 |
| Srsf5 | 7 |
| Timm50 | 7 |
| Idh3a | 7 |
| Mars | 7 |
| Hadhb | 7 |
| Farp1 | 7 |
| Rab35 | 7 |
| Arpc1b | 7 |
| Rpl34 | 7 |
| Atp6v1a | 7 |
| Eef1d | 7 |
| Rnd2 | 7 |
| Snrpa | 7 |
| Rangap1 | 7 |
| Emd | 7 |
| Arpc5l | 7 |
| Copb2 | 7 |
| Decr2 | 7 |
| Srsf7 | 7 |
| 1110051M20Rik | 7 |
| Rpl29 | 7 |
| G3bp1 | 7 |
| Spcs2 | 7 |
| Rars | 7 |
| Mapre2 | 7 |
| Aldh18a1 | 7 |
| Nav1 | 7 |
| Dhx30 | 7 |
| Fam120a | 7 |
| Arhgef11 | 7 |
| Nhsl1 | 7 |
| Rps24 | 6 |
| Rpl38 | 6 |
| Cox5a | 6 |
| Srsf2 | 6 |
| Rala | 6 |
| Marcks | 6 |
| Rpl37a | 6 |
| Caprin1 | 6 |
| Naca | 6 |
| Arpc5 | 6 |
| Pcbp1 | 6 |
| Dctn4 | 6 |
| Rdh11 | 6 |
| Rab18 | 6 |
| Psmd14 | 6 |
| Hnrnpd | 6 |
| Tmed10 | 6 |
| Rps28 | 6 |
| Ndufb10 | 6 |
| Dstn | 6 |
| Arl8a | 6 |
| Nup188 | 6 |
| Plp1 | 6 |
| Rpl22l1 | 6 |
| Nudt21 | 6 |
| Ndufb9 | 6 |
| Rpl36a | 6 |
| Atp6v0a1 | 6 |
| Sec61a1 | 6 |
| Srsf9 | 6 |
| Gpm6a | 6 |
| Gm10094 | 6 |
| Nup133 | 6 |
| Fubp3 | 6 |
| Crebbp | 6 |
| Uqcrfs1 | 6 |
| Pcdhga8 | 6 |
| Abtb2 | 6 |
| Amot | 6 |
| Myl6b | 6 |
| Mtap | 6 |
| Gap43 | 6 |
| Uggt1 | 6 |
| Ugt8a | 6 |
| Lin7c | 6 |
| Csde1 | 6 |
| Eef1g | 6 |
| Vapb | 6 |
| Jup | 6 |
| Cdc42bpb | 6 |
| Prkar2a | 6 |
| Aifm1 | 6 |
| Kif2a | 6 |
| Dock6 | 6 |
| Vti1b | 6 |
| Lars | 6 |
| Usp54 | 6 |
| Herc2 | 6 |
| Dnaja2 | 6 |
| Prkcq | 6 |
| Psmc5 | 6 |
| Smchd1 | 6 |
| Tubb5 | 5 |
| Rps27 | 5 |
| Gng12 | 5 |
| Rpl35 | 5 |
| Rab1b | 5 |
| Pcbp2 | 5 |
| mt-Co2 | 5 |
| Erh | 5 |
| H2afv | 5 |
| Pom121 | 5 |
| Syncrip | 5 |
| Serbp1 | 5 |
| Srsf10 | 5 |
| Gm5499 | 5 |
| Csnk2b | 5 |
| Eif3d | 5 |
| Rab21 | 5 |
| Uqcrq | 5 |
| Rplp2 | 5 |
| Zfp326 | 5 |
| Gm20716 | 5 |
| Fxr1 | 5 |
| Mlec | 5 |
| Decr1 | 5 |
| Ncam2 | 5 |
| Myh4 | 5 |
| Atp6v0d1 | 5 |
| Pdzd11 | 5 |
| Dctn3 | 5 |
| Snx18 | 5 |
| Gm4353 | 5 |
| Acat1 | 5 |
| Trio | 5 |
| Tnr | 5 |
| Stom | 5 |
| Kidins220 | 5 |
| Aimp2 | 5 |
| Scd2 | 5 |
| Ywhab | 5 |
| Ndufa13 | 5 |
| Dhrs7b | 5 |
| Slc25a22 | 5 |
| Pld1 | 5 |
| Pdia6 | 5 |
| Fam120c | 5 |
| Mar2 | 5 |
| Cope | 5 |
| Vamp3 | 5 |
| Por | 5 |
| Iars | 5 |
| Emc1 | 5 |
| Ipo5 | 5 |
| Chmp4b | 5 |
| Dsp | 5 |
| Slc4a4 | 5 |
| Rhot1 | 5 |
| Gm10116 | 5 |
| Lss | 5 |
| Trip12 | 5 |
| Srsf6 | 5 |
| Dnm2 | 5 |
| Cct5 | 5 |
| Sgpl1 | 5 |
| Psmc2 | 5 |
| Dync1i2 | 5 |
| Top2b | 5 |
| Ndc1 | 5 |
| Psma1 | 5 |
| Nap1l1 | 5 |
| Rap1gds1 | 5 |
| Lrrc49 | 5 |
| Map1a | 5 |
| Taok1 | 5 |
| Syne2 | 5 |
| Dynll2 | 4 |
| Atp5k | 4 |
| Hist3h2a | 4 |
| Dpy30 | 4 |
| Atp5l | 4 |
| Igkv8-28 | 4 |
| Ppp1cc | 4 |
| Gna11 | 4 |
| Ndufa8 | 4 |
| Snrpe | 4 |
| Cox6c | 4 |
| Cox5b | 4 |
| Gnai3 | 4 |
| Clasp2 | 4 |
| Grn | 4 |
| Elavl3 | 4 |
| Opcml | 4 |
| Rab5a | 4 |
| Erbb2ip | 4 |
| Gnai1 | 4 |
| Marcksl1 | 4 |
| Chtop | 4 |
| Rtn3 | 4 |
| Ndufa2 | 4 |
| Emc2 | 4 |
| Bax | 4 |
| 2700060E02Rik | 4 |
| Tomm22 | 4 |
| Eci1 | 4 |
| Snrpd3 | 4 |
| Myo9b | 4 |
| Hacd3 | 4 |
| Cct7 | 4 |
| Ddost | 4 |
| Stoml2 | 4 |
| Fis1 | 4 |
| Mapre1 | 4 |
| Gstp1 | 4 |
| Cyb5b | 4 |
| Psmc4 | 4 |
| Sf3b6 | 4 |
| Pde4b | 4 |
| Rbm3 | 4 |
| Ndufs7 | 4 |
| Ndufv2 | 4 |
| Puf60 | 4 |
| Hist1h1b | 4 |
| Ikbkap | 4 |
| Llgl1 | 4 |
| U2af2 | 4 |
| Ndufb4 | 4 |
| Mtx2 | 4 |
| Rhog | 4 |
| Eif5a | 4 |
| Cct6a | 4 |
| Stip1 | 4 |
| Smarcc2 | 4 |
| Srrm2 | 4 |
| Rpf2 | 4 |
| Ndufa12 | 4 |
| Gbas | 4 |
| Cyfip1 | 4 |
| Rras2 | 4 |
| Slc25a10 | 4 |
| Pfkm | 4 |
| Pgrmc1 | 4 |
| Psma4 | 4 |
| Cask | 4 |
| Ddah1 | 4 |
| Arpc1a | 4 |
| Srrt | 4 |
| Slc25a20 | 4 |
| Pebp1 | 4 |
| Psmc6 | 4 |
| Ptn | 4 |
| Ptges3 | 4 |
| G3bp2 | 4 |
| Idh3b | 4 |
| H2afx | 4 |
| Atp5d | 4 |
| Ctsb | 4 |
| Ube2v1 | 4 |
| Ndufa7 | 4 |
| Psma6 | 4 |
| Mpc2 | 4 |
| Cep170 | 4 |
| Cdc42bpa | 4 |
| Acadvl | 4 |
| Cbx3 | 4 |
| Vars | 4 |
| Prpf6 | 4 |
| Rab3a | 4 |
| Hspa4 | 4 |
| Ap2m1 | 4 |
| Asrgl1 | 4 |
| Arhgap23 | 4 |
| Ssb | 4 |
| Pcx | 4 |
| Etfb | 4 |
| Cenpv | 4 |
| Ncbp1 | 4 |
| Plekha6 | 4 |
| Cdk5 | 4 |
| Tomm34 | 4 |
| Synpo | 4 |
| Pura | 4 |
| Pbxip1 | 4 |
| Pgap1 | 4 |
| Nop16 | 4 |
| Gpd1l | 4 |
| Mrpl16 | 4 |
| Khdrbs1 | 4 |
| Trap1 | 4 |
| Tpt1 | 4 |
| Lmna | 4 |
| Aplp2 | 4 |
| Birc6 | 4 |
| Actg1 | 3 |
| Atp5j2 | 3 |
| Ndufa6 | 3 |
| Sec61b | 3 |
| Rplp1 | 3 |
| Nop56 | 3 |
| Pspc1 | 3 |
| Usmg5 | 3 |
| Sel1l | 3 |
| Uqcrc2 | 3 |
| Ssr4 | 3 |
| Gm5616 | 3 |
| Cdipt | 3 |
| Cand1 | 3 |
| Rps29 | 3 |
| Mmgt1 | 3 |
| Ilf2 | 3 |
| Ttn | 3 |
| Atp6v1e1 | 3 |
| Zfr | 3 |
| Rhob | 3 |
| Psmd8 | 3 |
| Pi4ka | 3 |
| Sep7 | 3 |
| Ptbp2 | 3 |
| Gm9843 | 3 |
| 2410015M20Rik | 3 |
| Smarcc1 | 3 |
| Tln1 | 3 |
| Ywhah | 3 |
| Tmem33 | 3 |
| Rab5b | 3 |
| Fxr2 | 3 |
| Mrps7 | 3 |
| Copb1 | 3 |
| Cisd1 | 3 |
| Abi1 | 3 |
| Snrpa1 | 3 |
| Psmb2 | 3 |
| Krt5 | 3 |
| Epdr1 | 3 |
| Hsd17b10 | 3 |
| Echs1 | 3 |
| Ank3 | 3 |
| Dad1 | 3 |
| Auh | 3 |
| Atp6v0c | 3 |
| Slc6a11 | 3 |
| Smpd4 | 3 |
| Tamm41 | 3 |
| Tmem55a | 3 |
| Ube2i | 3 |
| Tbc1d19 | 3 |
| Plekhb1 | 3 |
| Nicn1 | 3 |
| Rab8a | 3 |
| Mllt4 | 3 |
| Fhl1 | 3 |
| Gars | 3 |
| Arxes2 | 3 |
| Atad1 | 3 |
| Scarb2 | 3 |
| Ndufs8 | 3 |
| Hnrnph2 | 3 |
| Krt17 | 3 |
| Csrp1 | 3 |
| Bcas2 | 3 |
| Dlst | 3 |
| Rps21 | 3 |
| Txnl1 | 3 |
| Wdr6 | 3 |
| Slc25a29 | 3 |
| Timm17b | 3 |
| Tecpr1 | 3 |
| Nup62 | 3 |
| Nupl1 | 3 |
| Qk | 3 |
| Rbm8a | 3 |
| Rbm39 | 3 |
| Hmgb1 | 3 |
| Mrpl11 | 3 |
| Hmgcl | 3 |
| Arl8b | 3 |
| Tmed9 | 3 |
| Usp5 | 3 |
| Srrm1 | 3 |
| Thrap3 | 3 |
| Tmco1 | 3 |
| Pmvk | 3 |
| Numa1 | 3 |
| Pgls | 3 |
| Plekhg1 | 3 |
| Nebl | 3 |
| Mrpl1 | 3 |
| Mbd3 | 3 |
| Mib1 | 3 |
| Farsa | 3 |
| Mapk8ip3 | 3 |
| Dnajc13 | 3 |
| Cct4 | 3 |
| Csnk2a1 | 3 |
| Cox6b1 | 3 |
| C1qbp | 3 |
| Snx3 | 3 |
| Xpo1 | 3 |
| Tcp1 | 3 |
| Wasf1 | 3 |
| Uqcrb | 3 |
| Prps1l3 | 3 |
| Polr2b | 3 |
| Psat1 | 3 |
| Hk1 | 3 |
| Jak1 | 3 |
| Lrpprc | 3 |
| Kank1 | 3 |
| Hint1 | 3 |
| Cdc5l | 3 |
| Anp32e | 3 |
| Cap1 | 3 |
| Abhd17b | 3 |
| Ddx54 | 3 |
| Bcap31 | 3 |
| Shroom3 | 3 |
| Vcl | 3 |
| Safb2 | 3 |
| Pcmt1 | 3 |
| Myh2 | 3 |
| Prkcb | 3 |
| Myh11 | 3 |
| Ncald | 3 |
| Parp1 | 3 |
| Mycbp2 | 3 |
| Ranbp1 | 3 |
| Rpl37 | 3 |
| Psmc3 | 3 |
| Ppfibp1 | 3 |
| Rae1 | 3 |
| Glyr1 | 3 |
| Mpdu1 | 3 |
| Gstm5 | 3 |
| Hsd17b7 | 3 |
| Hnrnpll | 3 |
| Hnrnpul1 | 3 |
| Higd1a | 3 |
| Hnrnph3 | 3 |
| Khsrp | 3 |
| Dock1 | 3 |
| Sipa1 | 3 |
| Scp2 | 3 |
| Yme1l1 | 3 |
| Utrn | 3 |
| Wdfy3 | 3 |
| Plekha5 | 3 |
| Psmd9 | 3 |
| Qars | 3 |
| Polr2a | 3 |
| Prpf19 | 3 |
| Pdcd11 | 3 |
| Emc3 | 3 |
| Kdm1a | 3 |
| Itpr1 | 3 |
| Mrpl4 | 3 |
| Gpr17 | 3 |
| Mark3 | 3 |
| Hdlbp | 3 |
| Cs | 3 |
| Bclaf1 | 3 |
| Ccdc88a | 3 |
| Asna1 | 3 |
| Btf3 | 3 |
| Snrpd1 | 2 |
| Myl12a | 2 |
| Dynlt1a | 2 |
| Tusc3 | 2 |
| Tuba1b | 2 |
| Eif6 | 2 |
| Tomm20 | 2 |
| Lrrc57 | 2 |
| Rpl39 | 2 |
| Ndufb8 | 2 |
| Nxt1 | 2 |
| Ndufb7 | 2 |
| Eif3k | 2 |
| Krt42 | 2 |
| Magt1 | 2 |
| Tmed7 | 2 |
| Hist1h1e | 2 |
| 2010107E04Rik | 2 |
| Praf2 | 2 |
| Ogdh | 2 |
| Mrpl46 | 2 |
| Cacybp | 2 |
| Sccpdh | 2 |
| Rtn1 | 2 |
| Psma7 | 2 |
| Eif2s3x | 2 |
| Ankhd1 | 2 |
| Alg2 | 2 |
| Ap1s1 | 2 |
| Agpat5 | 2 |
| Tmem163 | 2 |
| Snrpf | 2 |
| Ndufa10 | 2 |
| Olig1 | 2 |
| Rap1b | 2 |
| Ctcf | 2 |
| Arf1 | 2 |
| Chst2 | 2 |
| Atp6v1g1 | 2 |
| Srp72 | 2 |
| Slc7a5 | 2 |
| Psma2 | 2 |
| Ndufa5 | 2 |
| Pfn2 | 2 |
| Igsf21 | 2 |
| Mcm5 | 2 |
| Lims1 | 2 |
| Gm10273 | 2 |
| Agpat1 | 2 |
| Sbf1 | 2 |
| Sf3b5 | 2 |
| Ubr4 | 2 |
| Psmd12 | 2 |
| Raly | 2 |
| Prnp | 2 |
| Psap | 2 |
| mt-Nd4 | 2 |
| Nf1 | 2 |
| Krt6a | 2 |
| Kif1bp | 2 |
| Gtpbp4 | 2 |
| Mrpl10 | 2 |
| Cdh13 | 2 |
| Calu | 2 |
| Zcchc24 | 2 |
| Ssr1 | 2 |
| Tra2b | 2 |
| Psen1 | 2 |
| Myl9 | 2 |
| Myo9a | 2 |
| Eif3i | 2 |
| Mlf2 | 2 |
| Fermt2 | 2 |
| Elovl1 | 2 |
| Fmr1 | 2 |
| Hist1h1a | 2 |
| Kcnj10 | 2 |
| Ddah2 | 2 |
| Cox6a1 | 2 |
| Yif1b | 2 |
| Tfrc | 2 |
| Sf3a1 | 2 |
| Stx7 | 2 |
| Srp68 | 2 |
| Unc79 | 2 |
| Rtn4 | 2 |
| Mtpn | 2 |
| Ppp1r12c | 2 |
| Npc1 | 2 |
| Eif3h | 2 |
| Eif4a3 | 2 |
| H1f0 | 2 |
| Hpx | 2 |
| Eif5b | 2 |
| Hmgb2 | 2 |
| Carhsp1 | 2 |
| Arf5 | 2 |
| Ddx50 | 2 |
| Comt | 2 |
| Tex10 | 2 |
| Ubxn1 | 2 |
| Sema3d | 2 |
| Timp3 | 2 |
| Rtcb | 2 |
| Tpr | 2 |
| Wdr36 | 2 |
| Runx2 | 2 |
| Spryd4 | 2 |
| Usp9x | 2 |
| Trim15 | 2 |
| Ndufb2 | 2 |
| Ppp2r2d | 2 |
| Psmb5 | 2 |
| Psmb1 | 2 |
| Rap1gap | 2 |
| Ndufb5 | 2 |
| Pds5b | 2 |
| Reep2 | 2 |
| Mgll | 2 |
| Mcu | 2 |
| Emc8 | 2 |
| Mlc1 | 2 |
| Gm20425 | 2 |
| Mrps26 | 2 |
| Ezr | 2 |
| Gtf3c1 | 2 |
| Fabp5 | 2 |
| Krt14 | 2 |
| Itgb1 | 2 |
| Esd | 2 |
| Eif3m | 2 |
| Dynlrb1 | 2 |
| Anp32b | 2 |
| Bri3bp | 2 |
| Apoo | 2 |
| Ednrb | 2 |
| Arhgap5 | 2 |
| Camk2g | 2 |
| Srpk2 | 2 |
| Ruvbl1 | 2 |
| Sec13 | 2 |
| Sar1a | 2 |
| Rps27l | 2 |
| Ubr3 | 2 |
| Spire1 | 2 |
| Snrpc | 2 |
| Snrpg | 2 |
| Sdhc | 2 |
| Snrpb2 | 2 |
| Pusl1 | 2 |
| Pisd | 2 |
| Prkra | 2 |
| Polr2i | 2 |
| Purb | 2 |
| Psmd4 | 2 |
| Nup85 | 2 |
| Nop2 | 2 |
| Nop10 | 2 |
| Rab33b | 2 |
| Ndufs6 | 2 |
| Phf5a | 2 |
| Prdx5 | 2 |
| Psmd13 | 2 |
| Glud1 | 2 |
| Mrps34 | 2 |
| Epb41l3 | 2 |
| Ik | 2 |
| Mrps23 | 2 |
| Exosc2 | 2 |
| Impa1 | 2 |
| Lonp1 | 2 |
| Gab1 | 2 |
| Mrpl14 | 2 |
| Ints3 | 2 |
| Erlin2 | 2 |
| Lrch1 | 2 |
| Kctd5 | 2 |
| Galc | 2 |
| Ict1 | 2 |
| Hepacam | 2 |
| Alpl | 2 |
| Ctsd | 2 |
| Atxn10 | 2 |
| Ccar2 | 2 |
| Ddx24 | 2 |
| Chchd6 | 2 |
| Cux1 | 2 |
| Eef1e1 | 2 |
| Ablim1 | 2 |
| Anapc1 | 2 |
| Afg3l2 | 2 |
| Crip2 | 2 |
| Cyb5a | 2 |
| Chmp6 | 2 |
| Actr1b | 2 |
| Cend1 | 2 |
| Atp6v1d | 2 |
| Cspg4 | 2 |
| Cttnbp2nl | 2 |
| Cdh4 | 2 |
| Dock3 | 2 |
| Srm | 2 |
| Soga3 | 2 |
| Rqcd1 | 2 |
| Rundc3a | 2 |
| Rraga | 2 |
| Zswim8 | 2 |
| Tet3 | 2 |
| Sbds | 2 |
| Tln2 | 2 |
| Ythdf2 | 2 |
| Smg1 | 2 |
| Vps35 | 2 |
| Zswim6 | 2 |
| Slc12a2 | 2 |
| Tomm70a | 2 |
| Tmem14c | 2 |
| Pdcd6 | 2 |
| Rai14 | 2 |
| Rgma | 2 |
| Ncapg2 | 2 |
| Ntm | 2 |
| Myh15 | 2 |
| Pbrm1 | 2 |
| Palld | 2 |
| Nt5dc2 | 2 |
| Psmd7 | 2 |
| Phgdh | 2 |
| Pan2 | 2 |
| Lypla2 | 2 |
| Kpna1 | 2 |
| Iws1 | 2 |
| Eln | 2 |
| Eif3l | 2 |
| Lamp1 | 2 |
| Faf2 | 2 |
| Elovl5 | 2 |
| Hyou1 | 2 |
| Gpd2 | 2 |
| H2-Ke6 | 2 |
| Gm6434 | 2 |
| Eif2s2 | 2 |
| Map4 | 2 |
| Fads2 | 2 |
| Kif7 | 2 |
| Etfdh | 2 |
| Cops7a | 2 |
| Daam1 | 2 |
| Cnbp | 2 |
| Cdh20 | 2 |
| Ctnnal1 | 2 |
| Cep131 | 2 |
| Acad11 | 2 |
| Acta1 | 2 |
| Cnn3 | 2 |
| Dync1li1 | 2 |
| Edrf1 | 2 |
| Atp10b | 2 |
| Ascc3 | 2 |
| Cpt1a | 2 |
| Ccar1 | 2 |
| Dars | 2 |
| Ddx18 | 2 |
| Cyth2 | 2 |
| Ccdc124 | 2 |
| Abcf1 | 2 |
| Ankfy1 | 2 |
| Cirbp | 2 |
