# Supplementary material for: PRMT5 Interacting Partners and Substrates in Oligodendrocyte Lineage Cells
Source: Front Cell Neurosci. 2022 Mar 17;16:820226. doi: 10.3389/fncel.2022.820226 (PMC8968030; doi:10.3389/fncel.2022.820226)
Supplement: Supplementary Table 2 — Ontology terms of PRMT5 interacting partners in oligodendrocyte lineage cells. Top 50 molecular functions of PRMT5 interactors in oligodendrocyte lineage cells. Gene ontology was performed using DAVID analysis. The GO terms and relative p-values are shown. [file Table_2.DOCX]

**Table 2.**

| **Term** | ***p*-value** |
| --- | --- |
| GO:0044822~poly(A) RNA binding | 3.73E-128 |
| GO:0003735~structural constituent of ribosome | 4.16E-47 |
| GO:0003779~actin binding | 4.87E-40 |
| GO:0098641~cadherin binding involved in cell-cell adhesion | 2.52E-39 |
| GO:0000166~nucleotide binding | 1.77E-38 |
| GO:0005515~protein binding | 5.50E-32 |
| GO:0003924~GTP binding and GTPase activity | 2.12E-17 |
| GO:0003774~motor activity | 1.33E-13 |
| GO:0017056~structural constituent of nuclear pore | 4.57E-13 |
| GO:0008137~NADH dehydrogenase (ubiquinone) activity | 7.68E-11 |
| GO:0005487~nucleocytoplasmic transporter activity | 5.40E-10 |
| GO:0051082~unfolded protein binding | 4.37E-09 |
| GO:0016887~ATPase activity | 3.65E-08 |
| GO:0008017~microtubule binding | 1.11E-06 |
| GO:0005516~calmodulin binding | 3.81E-06 |
| GO:0008092~cytoskeletal protein binding | 3.82E-06 |
| GO:0030898~actin-dependent ATPase activity | 4.88E-06 |
| GO:0004004~ATP-dependent RNA helicase activity | 6.54E-06 |
| GO:0046933~proton-transporting ATP synthase activity, rotational mechanism | 9.23E-06 |
| GO:0044325~ion channel binding | 1.91E-05 |
| GO:0000146~microfilament motor activity | 2.75E-05 |
| GO:0048365~Rac GTPase binding | 3.36E-05 |
| GO:0045296~cadherin binding | 4.97E-05 |
| GO:0019001~guanyl nucleotide binding | 6.83E-05 |
| GO:0003743~translation initiation factor activity | 7.49E-05 |
| GO:0051022~Rho GDP-dissociation inhibitor binding | 7.57E-05 |
| GO:0009055~electron carrier activity | 9.21E-05 |
| GO:0004579~dolichyl-diphosphooligosaccharide-protein glycotransferase activity | 1.02E-04 |
| GO:0043021~ribonucleoprotein complex binding | 1.16E-04 |
| GO:0031625~ubiquitin protein ligase binding | 1.21E-04 |
| GO:0031489~myosin V binding | 1.49E-04 |
| GO:0004386~helicase activity | 1.72E-04 |
| GO:0017025~TBP-class protein binding | 2.11E-04 |
| GO:0036402~proteasome-activating ATPase activity | 2.16E-04 |
| GO:0004298~threonine-type endopeptidase activity | 3.98E-04 |
| GO:0008139~nuclear localization sequence binding | 5.32E-04 |
| GO:0030507~spectrin binding | 5.34E-04 |
| GO:0004812~aminoacyl-tRNA ligase activity | 5.93E-04 |
| GO:0001664~G-protein coupled receptor binding | 5.96E-04 |
| GO:0031369~translation initiation factor binding | 6.98E-04 |
| GO:0003857~3-hydroxyacyl-CoA dehydrogenase activity | 9.09E-04 |
| GO:0003746~translation elongation factor activity | 0.0011543 |
| GO:0001948~glycoprotein binding | 0.0012929 |
| GO:0017048~Rho GTPase binding | 0.0015451 |
| GO:0031072~heat shock protein binding | 0.0015907 |
| GO:0005198~structural molecule activity | 0.0020907 |
| GO:0047391~alkylglycerophosphoethanolamine phosphodiesterase activity | 0.0022944 |
| GO:0015631~tubulin binding | 0.0024202 |
| GO:0015078~hydrogen ion transmembrane transporter activity | 0.0033172 |
| GO:0031683~G-protein beta/gamma-subunit complex binding | 0.00396 |
