# Supplementary material for: PRMT5 Interacting Partners and Substrates in Oligodendrocyte Lineage Cells
Source: Front Cell Neurosci. 2022 Mar 17;16:820226. doi: 10.3389/fncel.2022.820226 (PMC8968030; doi:10.3389/fncel.2022.820226)
Supplement: Supplementary Table 3 — Peptides (n = 307) with differential Rme2s in PRMT5 KD cells compared to EGFP controls. The first column shows the total number of peptides with symmetrically methylated arginine residues that were identified in the control cells as significantly different from the PRMT5 knockdown cells. The corresponding log2foldchange and p-values of the significant peptides are shown in the second and third column, respectively. [file Table_3.DOCX]

**Table 3.**

| **Peptides** | **log2FoldChange** | ***p*-value** |
| --- | --- | --- |
| Snrpb | -1.80205128 | 0.00E+00 |
| Snrpb | -2.050801525 | 1.11E-232 |
| Snrpd3 | -0.958341173 | 6.95E-149 |
| Snrpb | -1.359717177 | 2.94E-132 |
| Snrpd3 | -1.253951241 | 5.69E-117 |
| Hnrnph1 | -1.662295806 | 2.08E-95 |
| Hnrnph1 | -1.662295806 | 2.08E-95 |
| Hnrnph1 | -2.282437753 | 3.9E-89 |
| Hnrnph1 | -1.895097617 | 3.56E-86 |
| Fubp1 | -2.006482317 | 6.11E-78 |
| Hnrnph1 | -1.872195578 | 2.66E-72 |
| Hnrnph1 | -1.578427723 | 2.96E-63 |
| Hnrnph1 | -1.578427723 | 2.96E-63 |
| Fubp1 | -1.610794802 | 5.84E-63 |
| Hnrnph1 | -2.479882933 | 3.85E-61 |
| Cct7 | -1.780168922 | 1.83E-57 |
| Cct7 | -1.62922063 | 4.86E-57 |
| Hnrnpd | -1.495041342 | 1.13E-54 |
| G3bp1 | -1.944667826 | 2.26E-54 |
| G3bp1 | -1.944667826 | 2.26E-54 |
| Snrpd3 | -0.711204799 | 4.3E-53 |
| Hnrnpd | -1.498949865 | 1.59E-47 |
| Zfp326 | -2.407954369 | 2.15E-46 |
| Zfp326 | -2.407954369 | 2.15E-46 |
| Hnrnph1 | -2.060311925 | 1.11E-42 |
| Hnrnph1 | -1.627967487 | 1.19E-41 |
| Hnrnph1 | -1.642579672 | 1.38E-41 |
| Fubp1 | -2.395405943 | 6.97E-39 |
| Snrpb | -1.791364025 | 1.07E-38 |
| Snrpb | -1.791364025 | 1.07E-38 |
| Hnrnph1 | -1.466921202 | 7.28E-36 |
| Fus | -1.738004241 | 1.68E-34 |
| Lsm4 | -2.251891788 | 2.58E-33 |
| Hnrnph1 | -1.49829634 | 8.3E-33 |
| Snrpn | -1.500848764 | 1.95E-32 |
| Lsm4 | -2.204368786 | 8.07E-32 |
| Hnrnph1 | -1.79886791 | 6.2E-31 |
| Fus | -1.907962355 | 2.02E-30 |
| Khsrp | -1.903502099 | 2.98E-30 |
| Hnrnph1 | -1.832440927 | 1.24E-29 |
| Snrpn | -1.758055879 | 2.24E-27 |
| Lsm4 | -1.319400565 | 2.64E-27 |
| Lsm4 | -1.319400565 | 2.64E-27 |
| Coil | -2.123954575 | 4.86E-27 |
| Hnrnph1 | -2.299479498 | 3.94E-25 |
| Hnrnph1 | -1.807194596 | 6.3E-25 |
| Hnrnph1 | -1.807194596 | 6.3E-25 |
| Hnrnph2 | -1.444610622 | 8.63E-25 |
| Hnrnph2 | -1.444610622 | 8.63E-25 |
| Hnrnph1 | -1.915324327 | 2.21E-23 |
| Khsrp | -2.10464208 | 5.69E-22 |
| Hnrnph1 | -2.160209993 | 7.07E-22 |
| Lsm4 | -1.552730515 | 2.61E-20 |
| Lsm4 | -1.552730515 | 2.61E-20 |
| Cct7 | -1.712509173 | 9.27E-20 |
| Cct7 | -1.712509173 | 9.27E-20 |
| Lsm4 | -1.7380976 | 9.37E-20 |
| Lsm4 | -1.710365665 | 1.27E-19 |
| Zfp326 | -2.027894996 | 1.56E-19 |
| Hnrnph1 | -1.656440716 | 3.16E-18 |
| Hnrnph1 | -1.656440716 | 3.16E-18 |
| Cct7 | -0.941567301 | 1.16E-17 |
| Hnrnph1 | -2.560345728 | 6.38E-17 |
| Rbm26 | -2.052578494 | 1.32E-16 |
| Hnrnph1 | -2.05724657 | 2.2E-16 |
| G3bp1 | -1.681791461 | 5.49E-16 |
| Zfp326 | -1.928122647 | 7.54E-16 |
| Coil | -2.289686858 | 8.33E-16 |
| Hnrnph1 | -2.017787113 | 1.04E-15 |
| Khsrp | -1.412720545 | 4.73E-15 |
| Fam120a | -2.468718155 | 5.43E-15 |
| Cnbp | -1.415255532 | 1.53E-14 |
| Fubp1 | -2.86086951 | 1.61E-14 |
| Fubp1 | -2.888167857 | 3.68E-14 |
| Coil | -1.985844338 | 5.66E-14 |
| Cnbp | -1.396408812 | 7.7E-14 |
| Khsrp | -1.348451643 | 8.37E-14 |
| Khsrp | -1.348451643 | 8.37E-14 |
| Snrpb | -2.106522515 | 1.39E-13 |
| Snrpb | -2.106522515 | 1.39E-13 |
| Rbm26 | -1.8100613 | 2.18E-13 |
| Fam120a | -2.616404984 | 2.44E-13 |
| Rbm3 | -1.156769089 | 4.4E-13 |
| Rbm3 | -1.156769089 | 4.4E-13 |
| Snrpd3 | -0.662722735 | 6.04E-13 |
| Snrpd3 | -0.662722735 | 6.04E-13 |
| Snrpn | -1.087080796 | 6.04E-13 |
| Khdrbs1 | -2.279298732 | 1.24E-12 |
| Fus | -1.313108956 | 1.33E-12 |
| G3bp2 | -1.225382413 | 1.22E-11 |
| Hnrnph2 | -1.652665073 | 1.42E-11 |
| Hnrnph2 | -1.652665073 | 1.42E-11 |
| Nfxl1 | -1.661293653 | 1.68E-11 |
| Khdrbs1 | -2.666745812 | 3.22E-11 |
| Eif4g3 | -2.113861656 | 6.56E-11 |
| Hnrnpab | -0.9144176 | 1.95E-10 |
| Eif4g3 | -2.039310175 | 2.05E-10 |
| Aven | -1.44879488 | 2.6E-10 |
| G3bp1 | -1.804802241 | 3.31E-10 |
| Snrpb | -1.489223442 | 6.43E-10 |
| Snrpb | -1.967041302 | 6.69E-10 |
| Snrpb | -1.967041302 | 6.69E-10 |
| Nfxl1 | -1.653610489 | 9.23E-10 |
| Fus | -1.385242923 | 1.6E-09 |
| Hnrnpa1 | -0.97333904 | 6.18E-09 |
| Hnrnph2 | -1.175241164 | 6.87E-09 |
| Hnrnph2 | -1.175241164 | 6.87E-09 |
| G3bp2 | -1.316418193 | 7.22E-09 |
| Fus | -1.918360646 | 1.77E-08 |
| Coil | -2.429787017 | 2.21E-08 |
| Coil | -2.429787017 | 2.21E-08 |
| Hnrnpu | -0.446733143 | 2.51E-08 |
| Hnrnph1 | -1.158404994 | 2.54E-08 |
| G3bp1 | 3.311068832 | 3.41E-08 |
| G3bp1 | 3.311068832 | 3.41E-08 |
| G3bp1 | -1.481898943 | 3.55E-08 |
| Taf15 | -1.865054199 | 6.61E-08 |
| Hnrnph1 | -2.070247634 | 8.6E-08 |
| Hnrnph1 | -1.248258198 | 8.74E-08 |
| Hnrnph1 | -1.248258198 | 8.74E-08 |
| Hnrnpd | -1.468550241 | 1.15E-07 |
| Khdrbs2 | -2.132900915 | 1.85E-07 |
| Hnrnph1 | -1.821565319 | 2.24E-07 |
| Hnrnph1 | -1.821565319 | 2.24E-07 |
| Snrpd3 | -0.6355525 | 4.14E-07 |
| Khdrbs2 | -2.29715567 | 4.34E-07 |
| Prrc2c | -1.280162014 | 4.45E-07 |
| Prrc2c | -1.280162014 | 4.45E-07 |
| Snrpb | -2.021926775 | 4.81E-07 |
| Hnrnph1 | -2.159784069 | 5.4E-07 |
| Hnrnpa1 | -1.126508515 | 6.07E-07 |
| Snrpb | 0.211986334 | 6.87E-07 |
| Snrpb | 0.211986334 | 6.87E-07 |
| Snrpb | -2.10607618 | 6.92E-07 |
| Yrdc | -1.921055301 | 7.29E-07 |
| Yrdc | -1.921055301 | 7.29E-07 |
| Aven | -1.571678085 | 1.12E-06 |
| Fus | -1.570334632 | 1.18E-06 |
| H2afz | -0.4131133 | 1.21E-06 |
| H2afz | -0.4131133 | 1.21E-06 |
| Hnrnpu | -0.480301923 | 1.74E-06 |
| Taf15 | -1.365383957 | 1.86E-06 |
| Aven | -1.252181751 | 2.86E-06 |
| Wasl | -0.377685171 | 3.74E-06 |
| Snrpd3 | -0.57648246 | 4.96E-06 |
| Hnrnpab | -0.951355168 | 4.98E-06 |
| Zfp326 | -2.435381085 | 5.73E-06 |
| Fus | -1.381428462 | 7.18E-06 |
| Fus | -1.594115779 | 7.98E-06 |
| Fus | -1.594115779 | 7.98E-06 |
| Prrc2c | -2.224450639 | 8.67E-06 |
| Prrc2c | -2.224450639 | 8.67E-06 |
| Snrpb | 0.662243022 | 9.07E-06 |
| Snrpb | -1.386855991 | 1.03E-05 |
| Snrpb | -1.97292883 | 1.19E-05 |
| Srsf1 | -1.386895479 | 1.3E-05 |
| Srsf1 | -1.386895479 | 1.3E-05 |
| Fam98b | -2.28780543 | 1.5E-05 |
| Khdrbs2 | -2.113802387 | 1.52E-05 |
| Khdrbs2 | -2.113802387 | 1.52E-05 |
| Sfpq | -0.751859111 | 2.39E-05 |
| Khdrbs1 | -1.335570268 | 2.47E-05 |
| Khdrbs1 | -1.335570268 | 2.47E-05 |
| Hnrnph1 | -1.590250732 | 3.97E-05 |
| Hnrnph1 | -1.590250732 | 3.97E-05 |
| Fam120a | -1.557982711 | 4.14E-05 |
| Khdrbs1 | -1.407542802 | 5.06E-05 |
| Ewsr1 | -1.161647209 | 5.12E-05 |
| Ylpm1 | -1.922141968 | 5.17E-05 |
| Sfpq | -0.528999878 | 5.24E-05 |
| Fam98b | -4.107220014 | 5.66E-05 |
| Zfp326 | -2.086619281 | 6.39E-05 |
| Zfp326 | -2.086619281 | 6.39E-05 |
| Snrpd3 | 0.362632403 | 6.96E-05 |
| Hnrnpab | -0.87132377 | 7.5E-05 |
| Hnrnph2 | -1.494801589 | 8.9E-05 |
| Hnrnph2 | -1.494801589 | 8.9E-05 |
| Taf15 | -0.699156968 | 8.92E-05 |
| Hnrnph1 | -1.359323285 | 9.53E-05 |
| Hnrnph1 | -1.359323285 | 9.53E-05 |
| Snrpb | -2.256917509 | 0.000125594 |
| Snrpb | -2.256917509 | 0.000125594 |
| Zfp326 | -3.048804881 | 0.000148063 |
| Snrpb | -1.756768063 | 0.000163055 |
| Khdrbs1 | -2.07732563 | 0.000183895 |
| Khdrbs1 | -2.07732563 | 0.000183895 |
| Khdrbs1 | -2.414859136 | 0.000188031 |
| G3bp2 | -0.963654546 | 0.000194163 |
| G3bp1 | -2.37139524 | 0.000200683 |
| G3bp1 | -2.37139524 | 0.000200683 |
| Rbm26 | -1.034006279 | 0.000215851 |
| Virma | -2.531866103 | 0.000236476 |
| Hnrnph1 | -1.344141717 | 0.000242776 |
| Hnrnph1 | -1.344141717 | 0.000242776 |
| Snrpb | 0.474551673 | 0.000345156 |
| Snrpn | -1.616746835 | 0.00036548 |
| Snrpn | -1.616746835 | 0.00036548 |
| Lsm4 | -1.638617831 | 0.000398271 |
| Snrpb | -1.878486673 | 0.000459079 |
| Snrpb | -1.878486673 | 0.000459079 |
| Hnrnpa1 | -0.692528336 | 0.000472612 |
| Hnrnph1 | -1.510233042 | 0.000516695 |
| Snrpb | -1.615940972 | 0.000547486 |
| Snrpb | -1.615940972 | 0.000547486 |
| Mbp | -1.964424274 | 0.00068662 |
| Hnrnph1 | -1.630068647 | 0.000696788 |
| Snrpb | -2.055267536 | 0.000750156 |
| Snrpb | -2.055267536 | 0.000750156 |
| Zfp658 | -1.771133487 | 0.000758262 |
| Fus | -0.745501656 | 0.000803827 |
| Fubp1 | -3.272532657 | 0.000833726 |
| Fmr1 | -1.351208626 | 0.000863785 |
| Fmr1 | -1.351208626 | 0.000863785 |
| Wasl | -0.268378855 | 0.00090767 |
| Wasl | -0.674373744 | 0.000963126 |
| Wdr33 | -1.394398797 | 0.001230278 |
| Hnrnpa3 | -0.398398893 | 0.001307445 |
| Hnrnpa3 | -0.398398893 | 0.001307445 |
| Dpysl3 | -0.985675287 | 0.001349023 |
| Dpysl3 | -0.985675287 | 0.001349023 |
| Wdr33 | -1.653285601 | 0.001363154 |
| Thoc4 | -0.308574527 | 0.00140129 |
| Fmr1 | -0.916844408 | 0.001434737 |
| Fmr1 | -0.916844408 | 0.001434737 |
| Sfpq | -1.197577577 | 0.001607003 |
| Khsrp | -3.937948477 | 0.001634236 |
| Khsrp | -3.937948477 | 0.001634236 |
| Khdrbs2 | -1.971355722 | 0.001765556 |
| Pnn | -1.877884431 | 0.001857488 |
| Snrpb | -1.262793239 | 0.001907563 |
| Snrpb | -1.262793239 | 0.001907563 |
| Ylpm1 | -1.162572213 | 0.002010342 |
| Snrpd3 | -1.016900535 | 0.002016651 |
| Snrpd3 | -1.016900535 | 0.002016651 |
| Hnrnph2 | -1.19882077 | 0.002025182 |
| Hnrnph2 | -1.19882077 | 0.002025182 |
| Rps10 | -1.08458138 | 0.002059636 |
| Fam98b | -3.300110014 | 0.002095085 |
| Fam98b | -2.288560337 | 0.002235536 |
| Fubp1 | -3.593356022 | 0.002295569 |
| Hnrnph1 | -0.591554233 | 0.002320414 |
| Hnrnph1 | -0.591554233 | 0.002320414 |
| Prrc2c | -1.325316274 | 0.002332787 |
| Khsrp | -2.327892937 | 0.00247452 |
| Khsrp | -2.327892937 | 0.00247452 |
| Khdrbs1 | -1.02480035 | 0.002559245 |
| Hnrnpu | -0.420943646 | 0.002872965 |
| Hnrnpu | -0.420943646 | 0.002872965 |
| Prr3 | -1.22706549 | 0.003138743 |
| Prr3 | -1.22706549 | 0.003138743 |
| Snrpd3 | 0.590145984 | 0.003249043 |
| Khdrbs2 | -1.725946369 | 0.003277922 |
| Gigyf2 | -1.076006447 | 0.003311034 |
| Gigyf2 | -1.076006447 | 0.003311034 |
| Snrpb | -4.325416915 | 0.003905994 |
| Snrpb | -4.325416915 | 0.003905994 |
| Cdc42ep1 | -0.945816847 | 0.00407682 |
| Cdc42ep1 | -0.945816847 | 0.00407682 |
| Ndufs2 | -0.363011928 | 0.004157652 |
| Ndufs2 | -0.363011928 | 0.004157652 |
| Paip1 | -1.648254556 | 0.004168001 |
| Paip1 | -1.648254556 | 0.004168001 |
| Pnn | -2.030108404 | 0.004322791 |
| Hnrnpd | -1.42096744 | 0.004361031 |
| Rps10 | -1.373119454 | 0.00469967 |
| Hnrnpu | -0.391584434 | 0.004743922 |
| Prrc2c | -1.078522168 | 0.004907968 |
| Virma | -2.222117092 | 0.005643203 |
| Dhx9 | -2.318539967 | 0.005904673 |
| Dhx9 | -2.318539967 | 0.005904673 |
| Baz1a | 0.644425878 | 0.005930937 |
| Baz1a | 0.644425878 | 0.005930937 |
| Fmr1 | -1.25892347 | 0.006974489 |
| Fmr1 | -1.25892347 | 0.006974489 |
| Sf3b2 | 0.069875536 | 0.00766472 |
| Mbp | -1.954398327 | 0.00920402 |
| Fubp1 | -0.973087463 | 0.009713301 |
| Fubp1 | -0.973087463 | 0.009713301 |
| Zfp658 | -1.774062665 | 0.009750049 |
| Fam98b | -5.452403347 | 0.012192054 |
| Zfp326 | -1.569371021 | 0.012589118 |
| Thoc4 | -0.263583671 | 0.012696486 |
| Khdrbs2 | -2.810065089 | 0.012758623 |
| Khdrbs2 | -2.810065089 | 0.012758623 |
| Fam98b | -2.286861796 | 0.013067464 |
| Hnrnpk | -0.166878181 | 0.014891004 |
| Hnrnpk | -0.166878181 | 0.014891004 |
| Lsm4 | -0.777959004 | 0.016712373 |
| Dazap1 | -1.498059427 | 0.018175393 |
| Dazap1 | -1.498059427 | 0.018175393 |
| Hnrnpa0 | -0.332904685 | 0.019349003 |
| Hnrnpa0 | -0.332904685 | 0.019349003 |
| Nde1 | -1.488625433 | 0.026883081 |
| Nde1 | -1.488625433 | 0.026883081 |
| Khdrbs1 | -5.054237587 | 0.029604998 |
| Khdrbs1 | -5.054237587 | 0.029604998 |
| Hnrnph1 | -1.49838677 | 0.031030348 |
| Hnrnph1 | -1.49838677 | 0.031030348 |
| Snrpd3 | -0.812762621 | 0.034005824 |
| Sfpq | -1.379886317 | 0.036045765 |
| Snrpd3 | -1.732382805 | 0.040582423 |
| Sfpq | -1.531733587 | 0.041634689 |
| Rbm33 | -0.675833179 | 0.044710426 |
| Rbm33 | -0.675833179 | 0.044710426 |
| Thoc4 | -0.374330394 | 0.047298388 |
| Hnrnph1 | -0.44417987 | 0.047986264 |
| Hnrnph1 | -0.44417987 | 0.047986264 |
