# Supplementary material for: PRMT5 Interacting Partners and Substrates in Oligodendrocyte Lineage Cells
Source: Front Cell Neurosci. 2022 Mar 17;16:820226. doi: 10.3389/fncel.2022.820226 (PMC8968030; doi:10.3389/fncel.2022.820226)
Supplement: Supplementary Table 4 — Symmetrically dimethylated arginine residues of PRMT5 substrates in oligodendrocyte lineage cells. List of PRMT5 substrates, their Uniprot Protein Identification Codes and accession numbers, and the specific arginines residues identified as symmetrically dimethylated in the controls but not in PRMT5 knockdown cells. [file Table_4.DOCX]

**Table 4.**

| **Rme2  (*p*-value <0.05)** | **Uniprot Protein Identification Code** | **Uniprot Accession Number** | **Dimethylated Rs** |
| --- | --- | --- | --- |
| Aven | AVEN | Q9D9K3 | R70 |
| Baz1a | BAZ1A | O88379 | R193 |
| Cct7 | TCPH | P80313 | R536; R538 |
| Cdc42ep1 | BORG5 | Q91W92 | R53 |
| Cnbp | CNBP | P53996 | R25; R27; R30; R32; R34 |
| Coil | COIL | Q5SU73 | R446; R450 |
| Dazap1 | DAZP1 | Q9JII5 | R391 |
| Dhx9 | DHX9 | O70133 | R1365; R1376 |
| Eif4g3 | IF4G3 | Q80XI3 | R687 |
| Ewsr1 | EWS | Q61545 | R632; R635 |
| Fam120a | F120A | Q6A0A9 | R882; R884 |
| Fam98b | FA98B | Q80VD1 | R392; R398; R404; R410 |
| Fmr1 | FMR1 | P35922 | R470; R473 |
| Fubp1 | FUBP1 | Q91WJ8 | R354; R356; R358 |
| Fus | FUS | P56959 | R217; R219; R235; R237; R241; R244; R252; R387; R400 |
| G3bp1 | G3BP1 | P97855 | R445; R458 |
| G3bp2 | G3BP2 | P97379 | R468 |
| Gigyf2 | GGYF2 | Q6Y7W8 | R125; R127; R129; R131 |
| H2afz | H2AZ | P0C0S6 | R85 |
| Hnrnpa0 | ROA0 | Q9CX86 | R190; R193; R197; R199 |
| Hnrnpa1 | ROA1 | P49312 | R206; R218; R225 |
| Hnrnpa3 | ROA3 | Q8BG05 | R239; R246 |
| Hnrnpab | ROAA | Q99020 | R250 |
| Hnrnpd | HNRPD | Q60668 | R272; R278; R280; R282 |
| Hnrnph1 | HNRH1 | O35737 | R206; R212; R217; R224; |
| Hnrnph2 | HNRH2 | P70333 | R206; R212; R217; R224 |
| Hnrnpk | HNRPK | P61979 | R272; R275; R279 |
| Hnrnpu | HNRPU | Q8VEK3 | R696; R703; R709; R715 |
| Khdrbs1 | KHDR1 | Q60749 | R284; R289; R291; R302; R304; R310; R315; R340; R346 |
| Khsrp | FUBP2 | Q3U0V1 | R412; R414; R416; |
| Lsm4 | LSM4 | Q9CY46 | R87; R89; R101; R108; R114; R116; R124 |
| Nde1 | NDE1 | Q9CZA6 | R293 |
| Ndufs2 | NDUS2 | Q91WD5 | R118 |
| Nfxl1 | NFXL1 | E9Q8I7 | R63 |
| Paip1 | PAIP1 | Q8VE62 | R23 |
| Pnn | PININ | O35691 | R56 |
| Prr3 | PRR3 | Q811B5 | R101 |
| Prrc2c | PRC2C | Q3TLH4 | R280; R1159; R1161; R1163; R1169; R2689 |
| Rbm26 | RBM26 | Q6NZN0 | R855; R857 |
| Rbm3 | RBM3 | O89086 | R87; R90; R97; R99; R103 |
| Rbm33 | RBM33 | Q9CXK9 | R1091; R1099 |
| Rps10 | RS10 | P63325 | R158; R160 |
| Sf3b2 | SF3B2 | Q3UAI4 | R485 |
| Sfpq | SFPQ | Q8VIJ6 | R7; R9; R19; R25; R685 |
| Snrpb | RSMB | P27048 | R108; R112; R147; R172; R181; R196; R209 |
| Snrpd3 | SDM3 | P62320 | R97; R110; R112; R114; R118 |
| Snrpn | RSMN | P63163 | R108; R112 |
| Srsf1 | SRSF1 | Q6PDM2 | R97 |
| Taf15 | RBP56 | Q92804 | R183; R185 |
| Thoc4 | THOC4 | O08583 | R63; R196; R203 |
| Virma | VIR | A2AIV2 | R1802 |
| Wasl | WASL | Q91YD9 | R304 |
| Wdr33 | WDR33 | Q8K4P0 | R1256 |
| Ylpm1 | YLPM1 | Q9R0I7 | R1037 |
| Yrdc | YRDC | Q3U5F4 | R29 |
| Zfp326 | ZN326 | O88291 | R173; R175; R235; |
| Zfp658 | ZN658 | Q5TYW1 | R253 |
