# Supplementary material for: PRMT5 Interacting Partners and Substrates in Oligodendrocyte Lineage Cells
Source: Front Cell Neurosci. 2022 Mar 17;16:820226. doi: 10.3389/fncel.2022.820226 (PMC8968030; doi:10.3389/fncel.2022.820226)
Supplement: Supplementary Table 5 — Ontology terms of PRMT5 substrates in oligodendrocyte lineage cells. Molecular functions of PRMT5 substrates identified in cytosolic and nuclear extracts from oligodendrocyte lineage cells. Gene ontology was performed using DAVID analysis. The GO terms and relative p-values are shown. [file Table_5.DOCX]

**Table 5.**

Cytosolic substrates

| **Term** | ***p-*value** |
| --- | --- |
| GO:0044822~poly(A) RNA binding | 1.06E-29 |
| GO:0003723~RNA binding | 9.96E-22 |
| GO:0003676~nucleic acid binding | 2.01E-10 |
| GO:0000166~nucleotide binding | 3.31E-05 |
| GO:0003729~mRNA binding | 4.06E-04 |
| GO:0070034~telomerase RNA binding | 6.97E-04 |
| GO:0005515~protein binding | 7.59E-04 |
| GO:0071208~histone pre-mRNA DCP binding | 0.00737646 |
| GO:0003730~mRNA 3'-UTR binding | 0.00755095 |
| GO:0003677~DNA binding | 0.01264384 |
| GO:0030620~U2 snRNA binding | 0.01469976 |
| GO:0034046~poly(G) binding | 0.01712911 |
| GO:0003697~single-stranded DNA binding | 0.01779888 |
| GO:1990446~U1 snRNP binding | 0.01955261 |
| GO:0035925~mRNA 3'-UTR AU-rich region binding | 0.02918839 |
| GO:0008266~poly(U) RNA binding | 0.04346885 |
| GO:0008270~zinc ion binding | 0.0468354 |

Nuclear substrates

| **Term** | ***p-*value** |
| --- | --- |
| GO:0044822~poly(A) RNA binding | 3.65E-32 |
| GO:0003723~RNA binding | 3.66E-20 |
| GO:0003676~nucleic acid binding | 2.23E-12 |
| GO:0000166~nucleotide binding | 2.53E-07 |
| GO:0003729~mRNA binding | 1.53E-05 |
| GO:0003677~DNA binding | 4.88E-04 |
| GO:0070034~telomerase RNA binding | 5.73E-04 |
| GO:0071208~histone pre-mRNA DCP binding | 0.00669181 |
| GO:0030620~U2 snRNA binding | 0.01333998 |
| GO:1990446~U1 snRNP binding | 0.01774799 |
| GO:0035925~mRNA 3'-UTR AU-rich region binding | 0.02650651 |
