# Supplementary material for: PRMT5 Interacting Partners and Substrates in Oligodendrocyte Lineage Cells
Source: Front Cell Neurosci. 2022 Mar 17;16:820226. doi: 10.3389/fncel.2022.820226 (PMC8968030; doi:10.3389/fncel.2022.820226)
Supplement: Supplementary Table 6 — PRMT5 substrates in different cell lines. List of PRMT5 substrates identified in our study (immortalized oligodendrocyte progenitors), MiaPaca2 cells (Mulvaney et al., 2021), HeLa cells (Musiani et al., 2019), AML cells (Radzisheuskaya et al., 2019), and HEK293 cells (Li et al., 2021). Highlighted in red are substrates whose transcript levels are downregulated in PRMT5 KD cells (Scaglione et al., 2018). [file Table_6.DOCX]

**Table 6.**

|  | **Our data** | **Mulvaney *et al.,* 2021** | **Musiani *et al.,* 2019** | **Radzisheuskaya *et al.,* 2019** | **Li *et al*., 2021** |
| --- | --- | --- | --- | --- | --- |
| Wasl | X |  |  |  |  |
| Yrdc | X |  |  |  |  |
| Zfp658 | X |  |  |  |  |
| Nde1 | X |  |  |  |  |
| Mbp | X |  |  |  |  |
| H2afz | X |  |  |  |  |
| Dpysl3 | X |  |  |  |  |
| Dazap1 | X |  |  |  |  |
| Cdc42ep1 | X |  |  |  |  |
| Khdrbs2 | X |  |  |  |  |
| Prrc2c | X |  |  |  |  |
| Hnrnpd | X |  |  |  |  |
| Baz1a | X |  |  |  |  |
| Ndufs2 | X |  |  | X |  |
| Hnrnpa0 | X |  |  |  | X |
| Sf3b2 | X |  |  |  | X |
| Hnrnpu | X | X |  |  |  |
| Prr3 | X | X |  |  |  |
| Rbm33 | X | X |  |  |  |
| Ylpm1 | X | X |  |  |  |
| Virma | X | X |  |  |  |
| Aven | X |  |  | X |  |
| Eif4g3 | X | X |  |  |  |
| Fam98b | X | X |  |  |  |
| Lsm4 | X | X |  |  |  |
| Rbm26 | X | X |  |  |  |
| Snrpd3 | X | X |  |  |  |
| Alyref (Thoc4) | X |  |  | X |  |
| Srsf1 | X |  |  | X | X |
| G3bp2 | X | X |  | X |  |
| Hnrnpa3 | X | X | X |  |  |
| Hnrnpk | X | X |  |  | X |
| Hnrnpab | X | X | X |  |  |
| Fus | X | X |  |  | X |
| Khdrbs1 | X | X |  |  | X |
| Ewsr1 | X | X |  |  | X |
| Snrpn | X | X |  |  | X |
| Cnbp | X | X |  | X |  |
| Coil | X | X |  | X |  |
| Fmr1 | X | X |  | X |  |
| Hnrnph2 | X | X | X |  |  |
| Dhx9 | X | X | X |  |  |
| Paip1 | X | X | X | X |  |
| Pnn | X | X | X | X |  |
| Wdr33 | X | X | X | X |  |
| Taf15 | X | X |  | X | X |
| Cct7 | X | X |  | X | X |
| G3bp1 | X | X |  | X | X |
| Hnrnph1 | X | X | X |  | X |
| Fam120a | X | X | X | X |  |
| Khsrp | X | X | X | X |  |
| Sfpq | X | X | X | X |  |
| Znf326 | X | X | X | X |  |
| Gigyf2 | X | X | X | X | X |
| Fubp1 | X | X | X | X | X |
| Hnrnpa1 | X | X | X | X | X |
| Rbm3 | X | X | X | X | X |
| Snrpb | X | X | X | X | X |
| Rps10 | X | X | X | X | X |
| Rbm27 |  |  | X | X |  |
| Serbp1 |  | X | X | X |  |
| Hnrnph3 |  | X | X | X | X |
| Larp4b |  | X | X | X |  |
| Pspc1 |  | X | X | X | X |
| Srxn1 |  | X | X | X |  |
| Strbp |  | X | X | X |  |
| Supt5h |  | X | X | X |  |
| Larp4 |  | X | X | X |  |
| Atxn2 |  | X |  | X | X |
| Cpsf6 |  | X |  | X |  |
| Hnrnpa2b1 |  | X | X |  | X |
| Rbm47 |  | X | X |  |  |
| Srrt |  | X | X |  |  |
| Acin1 |  | X |  |  |  |
| Ccdc9 |  | X |  |  | X |
| Cirbp |  | X |  |  |  |
| Fam120c |  | X |  |  |  |
| Fubp3 |  | X |  |  |  |
| Fxr2 |  | X |  |  |  |
| Jmy |  | X |  |  |  |
| Krt19 |  | X |  |  |  |
| Msi2 |  | X |  |  |  |
| Ncl |  | X |  |  |  |
| Prpf6 |  | X |  |  |  |
| Rbfox2 |  | X |  |  |  |
| Btf3 |  |  | X | X |  |
| Ctage5 |  |  | X | X |  |
| Dync1li2 |  |  | X | X | X |
| Snx3 |  |  | X | X |  |
| Trip6 |  |  | X | X | X |
| Ccdc6 |  |  |  | X |  |
| Cct4 |  |  |  | X |  |
| Crip1 |  |  |  | X |  |
| Ddx3x |  |  |  | X |  |
| Dnajc13 |  |  |  | X |  |
| Eif4g1 |  |  |  | X | X |
| Flnb |  |  |  | X |  |
| Ilf3 |  |  |  | X |  |
| Irs2 |  |  |  | ~~X~~ |  |
| Pabpc4 |  |  |  | X | X |
| Pabpn1 |  |  |  | X |  |
| Rnaset2 |  |  |  | X |  |
| Rtca |  |  |  | X |  |
| Sart3 |  |  |  | X |  |
| Sec24c |  |  |  | X |  |
| Tubb6 |  |  |  | X |  |
| Vim |  |  |  | X |  |
| Vps13c |  |  |  | X |  |
| Was |  |  |  | X |  |
| Wipf1 |  |  |  | X | X |
| Zgpat |  |  |  | X |  |
| Akap8 |  |  | X |  |  |
| Ankrd17 |  |  | X |  |  |
| Atp5a1 |  |  | X |  |  |
| Proser2 |  | X | X |  |  |
| Cdc42bpb |  |  | X |  |  |
| Cgn |  |  | X |  |  |
| Cnot11 |  |  | X |  |  |
| Cps1 |  |  | X |  |  |
| Cul1 |  |  | X |  |  |
| Dcaf6 |  |  | X |  |  |
| Dcxr |  |  | X |  |  |
| Ddx17 |  |  | X |  | X |
| Dhx33 |  |  | X |  |  |
| Diaph1 |  |  | X |  |  |
| Dsp |  |  | X |  |  |
| Fbxl18 |  |  | X |  |  |
| Hnrnpdl |  |  | X |  | X |
| Kiaa1429 |  |  | X |  |  |
| Kif1c |  |  | X |  |  |
| Maged2 |  |  | X |  |  |
| Pkp3 |  |  | X |  |  |
| Plcl2 |  |  | X |  |  |
| Ppp1r13l |  |  | X |  |  |
| Rbmx |  |  | X |  |  |
| Safb |  |  | X |  |  |
| Svil |  |  | X |  | X |
| Wipf2 |  |  | X |  |  |
| Shisa8 |  |  |  |  | X |
| Arid1a |  |  |  |  | X |
| Syncrip |  |  |  |  | X |
| Scaf4 |  |  |  |  | X |
| Sec24a |  |  |  |  | X |
| Capns1 |  |  |  |  | X |
| Krt8 |  |  |  |  | X |
| Npm1 |  |  |  |  | X |
| Derpc |  |  |  |  | X |
| Srf |  |  |  |  | X |
| Slc25a4 |  |  |  |  | X |
| Slc25a6 |  |  |  |  | X |
| Ndufs1 |  |  |  |  | X |
| Rpl4 |  |  |  |  | X |
| Atrx |  |  |  |  | X |
| Ep300 |  |  |  |  | X |
| Bptf |  |  |  |  | X |
| Lman2 |  |  |  |  | X |
| Caprin1 |  |  |  |  | X |
| Plec |  |  |  |  | X |
| Inpp5j |  |  |  |  | X |
| Phlda2 |  |  |  |  | X |
| Kiaa1217 |  |  |  |  | X |
| Pdcd6ip |  |  |  |  | X |
| Gatad2b |  |  |  |  | X |
| Tfg |  |  |  |  | X |
| Smarcd1 |  |  |  |  | X |
| Nars2 |  |  |  |  | X |
| Qki |  |  |  |  | X |
| Dido1 |  |  |  |  | X |
| Xrn2 |  |  |  |  | X |
| Dhx36 |  |  |  |  | X |
| Znf703 |  |  |  |  | X |
| Mbnl1 |  |  |  |  | X |
| Mrps18a |  |  |  |  | X |
| Jcad |  |  |  |  | X |
| Atxn7l1 |  |  |  |  | X |
| Akap8l |  |  |  |  | X |
| Hnrnpul1 |  |  |  | X | X |
